# Enhancing acetate-based biosurfactant production by *Pseudomonas putida* KT2440 through fermentation optimization

**DOI:** 10.1186/s12934-026-03129-0

**Published:** 2026-09-29

**Authors:** Carina V. Michel, Christopher Weike, Laura N. Breuer, Luca A. I. Hampe, Miriam Schneider, Giancarlo Franciò, Walter Leitner, Till Tiso, Lars M. Blank

**Affiliations:** 1 Institute of Applied Microbiology – iAMB, Aachen Biology and Biotechnology – ABBt, RWTH Aachen University, Worringer Weg 1, 52074 Aachen, Germany; 2 Institut für Technische und Makromolekulare Chemie (ITMC), RWTH Aachen University, Aachen, Germany; 3 Max Planck Institute for Chemical Energy Conversion (MPI CEC), Mülheim an der Ruhr, Germany; 4 Systems Biotechnology, Faculty of Technology, Bielefeld University, Universitätsstr. 25, 33615 Bielefeld, Germany; 5 WSS center catalaix, Aachen, Germany

**Keywords:** Biosurfactants, HAAs, *Pseudomonas putida*, Feeding strategies, Fermentation design, Acetate, CO_2_-derived substrates

## Abstract

**Background:**

There is an increasing demand for sustainable chemicals, although “green premium” can be rarily achieved. In this context, biosurfactants emerge as promising substitutes for petroleum- or palm-oil-based surfactants due to their potentially lower environmental impact. 3-(3-hydroxyalkanoyloxy)alkanoic acids (HAAs) are biosurfactants composed of dimeric esterified hydroxy fatty acids. They serve as precursor molecules for rhamnolipids. Here, acetate was chosen as potentially CO_2_-derived carbon source to replace conventional sugar-based feedstocks for HAA production using the versatile bacterium *Pseudomonas putida* KT2440 as heterologous production host.

**Results:**

In this study, a fed-batch fermentation for microbial HAA production with acetate as sole carbon source was developed and optimized. First, different pH values were evaluated to determine optimal cultivation conditions, and a pH of 7.8 was identified as the optimal setpoint. To enhance the overall productivity of the fermentation process, different feeding strategies were investigated. Acetate feeding was controlled either by dissolved oxygen (DO)-triggered substrate addition or by pH-coupled feeding using acetic acid as titrant and carbon source. Both feeding strategies resulted in periods of carbon limitation, which promoted HAA degradation. To overcome this challenge, urea was investigated as an alternative nitrogen source in the pH-coupled feeding strategy. Here, the highest reported HAA concentration of 1 g L⁻¹ was achieved, with more than 3.5-fold increases in space-time and product-to-substrate yields compared to the ammonium-based pH-coupled feeding. As a next step, the implementation of CO_2_-derived acetate as carbon source was tackled to improve the overall carbon footprint of the bioprocess. To this end, different substrate mixtures obtained from chemocatalytic CO_2_ conversion were evaluated for biocompatibility, growth, and HAA production. The results demonstrate the suitability of the CO_2_-derived acetate-formate mixture for the bioprocess without purification, achieving growth and HAA production comparable to the reference.

**Conclusion:**

This study demonstrates the successful establishment of a bioprocess for heterologous production of HAAs using acetate as carbon source. Urea as alternative nitrogen source not only enhanced process performance and simplified process control but also improved the overall sustainability of the fermentation process. Finally, the successful valorization of CO_2_-derived acetate further enhances its role as a next-generation feedstock in an emerging bioeconomy.

**Supplementary Information:**

The online version contains supplementary material available at https://doi.org/10.1186/s12934-026-03129-0.

## Introduction

**Fig. 1:**
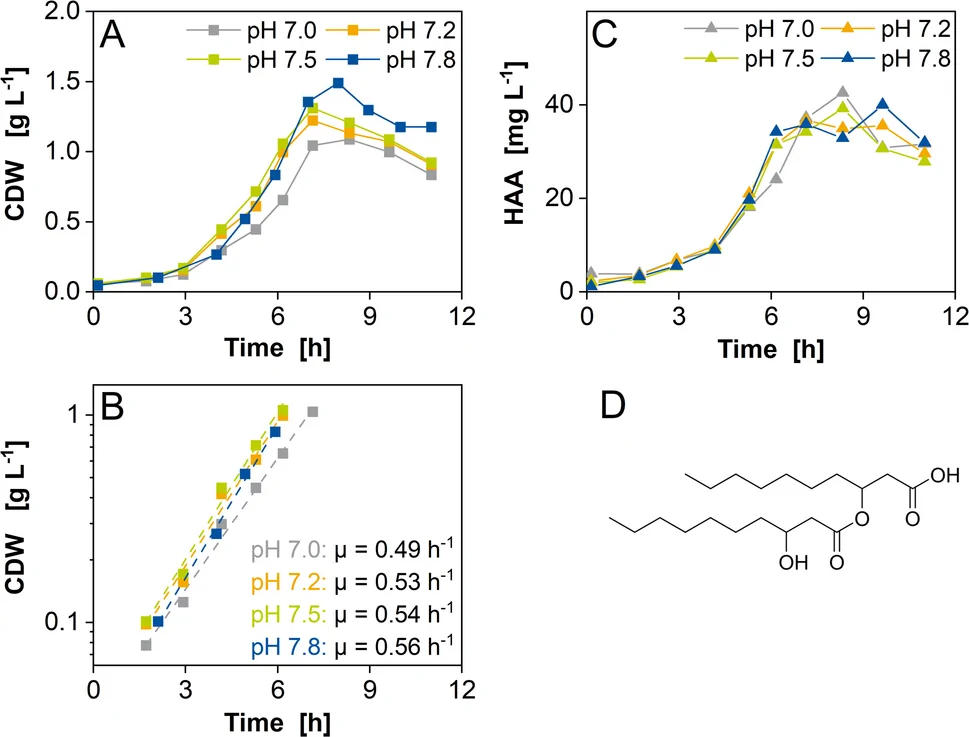
Investigation of pH on growth and HAA formation using *P. putida* TALE_*rhlA*. **A** Biomass (squares), **B** specific growth rates, **C** HAA concentrations (triangles) over time, and **D** molecular structure of HAAs (C_10_-C_10_ fatty acid chains). Cultivation conditions: MSM medium containing 5 g L^− 1^ acetate, nitrogen source: ammonium sulfate, 1.3 L stirred-tank bioreactors with 500 mL filling volume, T = 30 °C, *N* = 400–1,000 rpm (cascaded to maintain a DO ≥ 30%), F_Air_ = 0.5 L min^− 1^, OD_start_ = 0.2, pH values: 7.0, 7.2, 7.5, and 7.8, pH values maintained by adding either 4 M KOH or 4 M H_2_SO_4_. CDW: cell dry weight, DO: dissolved oxygen

Biosurfactants are amphiphilic molecules of microbial origin that consist of a hydrophilic head group and a hydrophobic tail [1]. Compared to their petroleum-based counterparts, whose production is energy-intensive and relies on harsh reaction conditions [2], biosurfactants are produced naturally from renewable feedstocks, resulting in compounds with lower toxicity, enhanced biodegradability, and improved overall environmental compatibility [3–5]. 3-(3-hydroxyalkanoyloxy)alkanoic acids (HAAs) serve as natural precursors in the metabolic pathway for rhamnolipids [6, 7]. Structurally, HAAs consist of two hydroxy fatty acids linked by an ester bond (Fig. 1), which are produced as mixtures of different congeners that combine saturated and unsaturated fatty acids and chain lengths ranging from four to 18 carbon atoms [8]. Due to their amphiphilic structure, HAAs show surface-active properties. Besides their application as biosurfactants in the chemical industry, HAAs have potentially a broad application spectrum, ranging from biochemical building blocks to emulsifiers [9, 10]. Moreover, HAAs can be incorporated into partially biobased poly(amide-urethanes) *via* co-polymerization, thereby enabling bioplastic production [11]. Their chemical structure also renders them promising candidates for further conversion into biofuels [12, 13].

The biosynthesis of HAAs occurs naturally in the pathogenic organism *Pseudomonas aeruginosa*. The precursor 3-hydroxyacyl-ACP is synthesized in the fatty acid *de novo* synthesis [14, 15]. Subsequently, the enzyme acyltransferase A (RhlA, encoded by the gene *rhlA*), catalyzes the condensation reaction between two β-hydroxyacyl-ACPs to form HAAs [7]. The congener mixture produced is dependent on variations within the RhlA enzyme, with C_10_-C_10_ being the main congener when utilizing *rhlA* from *P. aeruginosa* PAO1 [6, 16]. Despite its role as an efficient native production host for HAAs, *P. aeruginosa* presents several disadvantages, including pathogenicity and complex regulatory mechanisms, making its industrial use difficult [15, 17, 18]. Additionally, it prefers hydrophobic substrates such as vegetable oils, which complicate downstream processing [19]. To overcome these challenges and facilitate safe production methods, heterologous HAA expression systems have been successfully established using non-pathogenic microorganisms, such as *Pseudomonas putida* KT2440, *via* stable genetic integration of the *rhlA* gene originating from *P. aeruginosa* PAO1, combined with strong constitutive promoters [9, 20–23]. *P. putida*, a Gram-negative soil bacterium, is a well-established chassis in industrial biotechnology [24, 25]. It is known to naturally utilize a wide range of carbon sources, including sugars, fatty acids, aromatic hydrocarbons, and organic acids such as acetate [26–28]. Moreover, the strain was successfully engineered to metabolize xylose and other sugars derived from lignocellulosic biomass as well as plastic monomers [29–31]. Additionally, *P. putida* can utilize formate as an energy source for enhanced reduction equivalent regeneration, leading to increased biomass yields [32]. Moreover, the organism is characterized by its versatile nitrogen metabolism, ranging from ammonium and nitrate to various amino acids and urea [33, 34].

To support the transition towards a circular bioeconomy, there is an increasing need to replace first-generation feedstocks such as glucose and other sugar-based carbon sources, which are still widely used in biotechnological processes, to avoid competition with the food and feed sector [35]. In this context, the C2 compound acetate arises as potentially CO_2_-derived carbon source as it can be synthesized *via* different sustainable production routes, as reviewed by Lim, Lee [36] and Novak and Pflügl [37]. These routes include depolymerization of lignocellulosic biomass [38], the use of C1 gases in syngas fermentation with acetogenic bacteria [39–42], microbial electrosynthesis, and methane oxidation [43–46]. Moreover, chemical catalysis methods have been explored. For instance, the isomerization of CO_2_-derived methyl formate to acetic acid and the catalytic synthesis of acetic acid from methanol using formic acid as a renewable CO source have been proposed as alternative routes [47–49]. Acetate is water-soluble and non-viscous, making it easy to use as carbon source without mass-transfer issues. Moreover, its uptake increases the pH, thereby enabling pH regulation through acetic acid addition and serving as an online parameter for pH-coupled feeding strategies [50–52]. At the cellular level, acetate uptake occurs through either facilitated diffusion over the membrane in its undissociated form or active transport under energy consumption in its dissociated form [53–55]. Once inside the cell, acetate is converted into acetyl-CoA, which serves as a direct precursor for various metabolic pathways, including the TCA cycle, the glyoxylate cycle, and fatty acid synthesis [56, 57]. These pathways contribute to the production of secondary metabolites and other bioproducts [57, 58]. Research shows that various organisms, such as *P. putida*, *Escherichia coli*, and *Corynebacterium glutamicum*, can tolerate and utilize at least 5 g L^− 1^ acetate as their sole carbon source [28, 59–62] and produce various bioproducts including organic acids, such as succinic acid and itaconic acid, or rhamnolipids from acetate as sole carbon source [51, 59, 61, 63]. However, high acetate concentrations induce weak acid stress, resulting in cytosolic acidification and growth inhibition [57]. Therefore, most acetate-based bioprocesses remain less competitive than their sugar-based counterparts. Given the inhibitory effects of high acetate concentrations, effective feeding strategies are crucial for successful bioproduct synthesis from acetate during fed-batch fermentations.

Although microbial biosurfactant production aligns with the global shift towards sustainable and green technologies [64, 65], its production *via* fermentation encounters significant challenges, particularly massive foaming, due to its surface-active properties [66, 67]. This foaming issue can hinder efficient and scalable production processes [68]. Foam-related problems in bioprocesses include loss of biocatalysts, oxygen and nutrient limitations, and disruptions in mass balance due to biomass loss [69–71]. Several solutions have been proposed to tackle foaming in biosurfactant production, including the addition of antifoaming agents [72], mechanical foam breakers [73, 74], in situ extraction [75, 76], foam fractionation [77–79], surface aeration under pressurized conditions [66], or defoaming molecules as substrates [80, 81]. An innovative approach is membrane aeration, which offers a bubble-free alternative by allowing oxygen to diffuse through a membrane, thereby avoiding foam formation [82, 83]. This technique eliminates the need for antifoam for foam mitigation while maintaining comparable process performance to bubble-aerated systems [52].

In recent years, the use of co-substrates and feedstocks derived directly from carbon dioxide has gained increasing interest. This so-called “Power-to-X” strategy aims to convert renewable electricity into chemical energy carriers, enabling the sustainable synthesis of C1 and C2 compounds, such as methanol, formate, and acetic acid, directly from CO_2_ and green hydrogen [47–49]. Here, formate represents a promising substrate, as it can easily be generated from CO_2_ *via* catalytic hydrogenation [48]. Recent advances include biphasic catalytic systems employing ruthenium-based catalysts and various inorganic and organic stabilizing bases [84–86]. Since *P. putida* KT2440 can metabolize formate as an energy source, resulting in enhanced biomass yields [32], formate serves as an interesting co-substrate [87, 88]. Several feeding strategies using formate as co-substrate have been successfully developed in eukaryotic systems [89–91]. Ullmann, Guntermann [89] demonstrated the direct use of CO_2_-derived formate as a co-substrate, without further processing, in their glucose-based fermentation process for itaconate production using Ustilaginaceae.

In this study, a fermentation process for the heterologous production of HAAs with acetate as the sole carbon source was developed and optimized. First, optimal growth conditions for *P. putida* in batch fermentations were identified. As a next step, fed-batch operation was investigated by applying different feeding strategies to enhance titers, yields, and rates. Implementing urea as an alternative nitrogen source should not only improve process performance and simplify process control but also enhance the overall sustainability of the fermentation. To reduce the bioprocess’s carbon footprint, CO_2_-derived acetic acid and various CO_2_-derived acetate-formate mixtures were evaluated for biocompatibility, growth, and HAA production.

## Results and discussion

### High pH facilitates optimal growth on acetate

Bioprocess development in shake flasks with acetate as carbon source is challenging due to increasing pH values during acetate uptake and its inhibitory effect at higher concentrations. Therefore, to identify optimal growth and HAA production conditions of *P. putida* KT2440, four batch fermentations at constant pH values (7.0, 7.2, 7.5, 7.8) with an initial acetate concentration of 5 g L^− 1^ were performed. To avoid foam formation, surface aeration was applied. For these experiments, an acetate-tolerant derivative of *P. putida* KT2440 obtained by tolerance adaptive laboratory evolution (TALE) [92, 93], generated previously was used. The strain was evolved by serial cultivation under gradually increasing acetate concentrations, resulting in improved growth on acetate, including a reduced lag phase and enhanced tolerance towards elevated acetate concentrations compared to the parental wild-type strain [94]. The evolved strain was subsequently equipped with the heterologous *rhlA* gene for HAA production, yielding *P. putida* TALE_*rhlA* [94].

Applying higher pH values for cultivation influenced the growth of *P. putida* TALE_*rhlA* compared to the reference cultivation at pH 7.0 (Fig. 1). Increasing the pH value resulted in higher final biomass concentrations of up to 1.5 g L^− 1^ (pH 7.8) compared to 1.05 g L^− 1^ for pH 7.0 and specific growth rates ranging from 0.49 h^− 1^ at pH 7.0 to 0.56 h^− 1^ at pH 7.8, indicating a reduced inhibitory effect of acetate at higher pH values (Fig. 1A, B). At higher pH values, acetate predominantly exists in its dissociated form, thereby reducing the amount of the uncharged, more hydrophobic protonated form of acetic acid, which can enter the cells across the membrane *via* diffusion. Consequently, as pH increases, acetic acid uptake into the cell is limited, reducing weak acid stress and inhibitory effects and thereby improving growth performance [57]. Formation of HAAs remains stable across all tested pH values, ranging from 35 to 42 mg L^− 1^ at the end of the cultivation (Fig. 1C). However, despite the use of surface aeration, the addition of an antifoam agent was required. At high biomass concentrations toward the end of cultivation, increased agitation rates were required to meet the elevated oxygen demand, leading to the formation of small gas bubbles in the cultivation broth and ultimately to persistent foam formation.

The results demonstrate the suitability of acetate as next-generation feedstock. The results are in line with observations by Arnold [59], who reported rhamnolipid synthesis from lignocellulose-derived acetate, while Widberger [95] combined *Acetobacter*-produced acetate with rhamnolipid production. However, while these studies were conducted in shake flasks, the present study focuses on developing and optimizing fed-batch fermentation strategies for HAA production in bioreactors. Based on the optimized batch fermentation, subsequent investigations examine feeding strategies to enhance process performance.

### Exploring feeding strategies to enhance product titers and process performance

As a next step, different feeding strategies were applied to increase product titers and enhance process performance. To avoid foam formation, the membrane stirrer system developed by Bongartz [83], combining bubble-free membrane aeration and stirring, was used for all fed-batch fermentation experiments.

#### Carbon-limiting DO-based feeding strategy leads to HAA degradation

First, a dissolved oxygen (DO)-based, carbon-limiting feeding strategy was investigated [52, 75]. The fermentation was performed with a pH-neutral feeding solution containing 200 g L^− 1^ sodium acetate, while the batch medium initially contained 2.5 g L^− 1^ acetate. Following acetate depletion, indicated by an increase in the DO signal above 70%, acetate feeding was initiated at a constant flow rate of 43 mL h^− 1^. Feeding was maintained until the DO signal decreased to 40%, at which point the feed pump was switched off. Upon renewed acetate depletion, the DO signal increased again to 70%, triggering the next feeding cycle. Thus, the amount of acetate supplied per feeding cycle was not predefined but depended on the duration of pump activation, which was determined by the DO response. Consequently, acetate was supplied in a series of DO-dependent feed pulses throughout the cultivation. To prevent oxygen limitation, the oxygen supply was manually increased whenever the DO dropped below 15%.

**Fig. 2:**
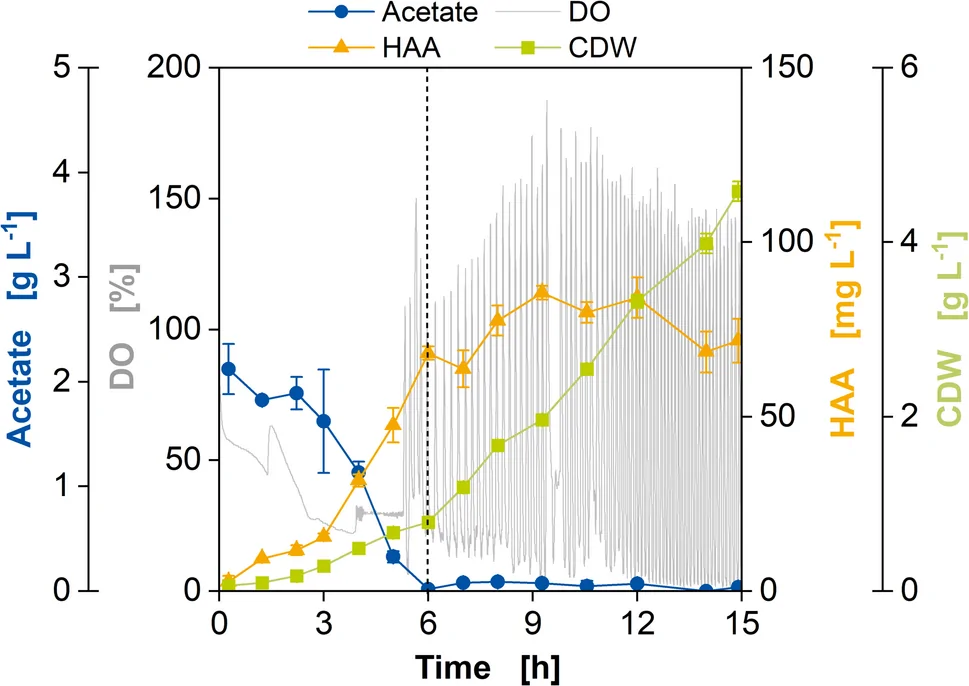
DO-based feeding strategy for enhanced HAA production. Membrane-aerated fed-batch cultivation with *P. putida* TALE_*rhlA*. Acetate concentration (blue line, circles), DO (grey lines), HAA concentration (orange line, triangles), and cell dry weight (green lines, squares) over time. Cultivation conditions: MSM medium containing 2.5 g L^− 1^ acetate (batch phase), and 1x buffer, nitrogen source: ammonium sulfate, 3 L stirred-tank bioreactors with 2 L filling volume, T = 30 °C, *N* = 100–350 rpm (cascaded to maintain a DO ≥ 30% during the batch phase with subsequent increase of transmembrane pressure (TMP) to 0.3 bar and supplementation of pure oxygen (21% − 100%) during the feeding phase), F_Air_ = 1 L min^− 1^, OD_start_ = 0.2, pH = 7.8, pH value maintained by adding either 4 M KOH or 4 M H_2_SO_4_. The feed solution contained concentrated medium components according to Sect.  4.1, m_Feed total_ = 15 ± 1 g acetate, CDW: cell dry weight, DO: dissolved oxygen. Fermentation was conducted in duplicates (*n* = 2). Error bars represent the standard deviation of the mean

After six hours of cultivation, acetate was entirely consumed, leading to a rise in dissolved oxygen (DO) that signaled the start of the feeding phase (Fig. 2). At the end of the batch phase, biomass reached 0.8 ± 0.1 g L^− 1^ CDW corresponding to a specific growth rate of 0.57 ± 0.02 h^− 1^. During the batch phase, 68 ± 2 mg L⁻¹ HAAs were produced (Fig. 2). Membrane aeration effectively prevented foam formation, eliminating the need for antifoam. During the batch phase, the DO was controlled and maintained at 30% *via* an agitation cascade, followed by the addition of pure oxygen (21% − 100%). After 2.5 h of cultivation, the DO rose to 45% due to the increase in transmembrane pressure to 0.3 bar. During the feeding phase, the DO-dependent control resulted in feed pulses of variable duration and acetate amount, as reflected by the oscillating DO signal. Biomass continued to increase linearly, reaching a final concentration of 5.0 ± 0.1 g L⁻¹ after 17 h of cultivation. Interestingly, HAA production peaked after 9 h and declined during the rest of the cultivation, with a maximal concentration of 82 ± 5 mg L⁻¹ (Fig. 2), corresponding to a Y_P/S_ of 11 ± 1 mg g^− 1^, and a STY of 9 ± 1 mg L^− 1^ h^− 1^ (Table 1). As HAA production is assumed to be growth-coupled due to its dependence on sigma70 promoters, which are active only during growth [20, 21], the observed HAA degradation during the feeding phase may be attributed to carbon limitation during that phase. Throughout the feeding phase, acetate levels remained consistently low, resulting in carbon-limited conditions. Therefore, carbon levels were assumed to be insufficient to support efficient HAA synthesis, even though biomass continued to increase (Fig. 2). Although a potential oxygen limitation was observed during the last three hours of cultivation (indicated by a DO being lower than 5% during the feeding events), it should not affect HAA production. In studies conducted by Weiser [66], HAAs were produced under oxygen-limited conditions, indicating that HAA stability is primarily determined by carbon availability rather than oxygen supply. HAA degradation has already been observed in studies by Tiso [9] and Wittgens [96], in which the authors assumed the cleavage of HAAs to 3-hydroxyalkanoic acids and subsequent uptake by *P. putida* under carbon-limited conditions. The fatty acids are metabolized *via* β-oxidation to form acetyl-CoA and ultimately ATP, a pathway that has already been described for the metabolization of intracellular polyhydroxyalkanoates (PHAs) in *P. putida* under starvation conditions, a storage molecule that shares the same precursor as HAAs [97].

In conclusion, the results demonstrate that the DO-based feeding strategy with an oscillating DO due to the DO dependent feeding events is not suitable for enhanced HAA production due to insufficient carbon availability during the starvation phases between the feeding events, resulting in HAA degradation. These findings emphasize the need for an optimized feeding strategy. Here, a pH-coupled feeding approach was investigated, which is known for its self-regulating kinetics.

**Table 1:** Performance indicators of conducted fed-batch fermentations with different feeding strategies

Strain		DO-based feeding (NH_4_ sulfate)	pH-coupled feeding (NH_4_ sulfate)	DO- and pH-coupled feeding (NH_4_ sulfate)	Optimized pH-coupled feeding (Urea)
		*. putida* TALE_*rhlA*	P*. putida * TALE_*rhlA*	P*. putida * KS3	P*. putida * KS3
Time	[h]	9	10	17	21
µ_max, Batch_	[h^− 1^]	0.57 ± 0.01	0.61	0.49	0.45 ± 0.02
µ_Feed phase_	[h^− 1^]	0.02 ± 0.01	0.02	0.16	---
m_Acetate fed_	[g]	15 ± 1	16	57	47 ± 1
c_max, HAA_	[mg L^− 1^]	82 ± 5	130	545	1,080 ± 115
Y_X/S_	[g_CDW_ g_Ac_^−1^]	0.27 ± 0.01	0.32	0.22	0.23 ± 0.01
Y_P/S, used_	[mg_HAA_ g_Ac_^−1^]	11 ± 1	16	19	48 ± 4
Y_P/X_	[mg_HAA_ g_CDW_^−1^]	42 ± 4	47	86	205 ± 17
STY	[mg_HAA_ L^− 1^ h^− 1^]	9 ± 1	13	31	51 ± 5

*P. putida* TALE_*rhlA*: *P. putida* KT2440 evolved for acetate tolerance equipped with gene for HAA production, *P. putida* KS3: *P. putida* KT2440 wild-type strain equipped with gene for HAA production, Time = cultivation time with maximal HAA concentration, µ_max, Batch_ = specific growth rate in batch phase, µ_Feed phase_ = specific growth rate during feeding phase, m_Acetate, fed_ = total amount of acetate fed during cultivation, c_HAA, max_ = maximal HAA concentration at the end of cultivation, Y_X/S_ = Biomass-to-substrate yield, Y_P/S_ = Product-to-substrate yield, Y_P/X_ = Product-to-biomass yield, STY = Space-time-yield. pH-coupled feeding and DO- and pH-coupled feeding with ammonium sulfate were conducted as inividual experiments (*n* = 1); therefore, no standard deviations could be calculated. DO-based feeding and pH-coupled urea feeding were conducted in duplicates (*n* = 2). Error bars represent the standard deviation of the mean.

#### pH-coupled feeding strategy is insufficient to maintain adequate levels of acetate

To overcome the carbon limitation and HAA degradation observed during DO-based feeding, a pH-coupled feeding strategy was implemented. The basis of this approach is the understanding that acetate consumption increases culture pH. In the pH-coupled feeding approach, this elevation triggers the addition of glacial acetic acid, thereby lowering the pH and simultaneously serving as a carbon source. This creates a self-regulating system that automatically adjusts feeding according to microbial growth kinetics [50–52]. In theory, the feed rate should be linked to acetate uptake [50], thereby preventing carbon limitation and associated HAA degradation. Furthermore, this approach minimizes dilution effects due to using highly concentrated feedstocks, thereby keeping the reactor filling volume constant. Moreover, overall salt loads are lower than those with sodium acetate-based feeding, making it a promising strategy for sustaining higher, more stable HAA production throughout cultivation [52]. The fed-batch fermentation experiment was conducted with 5 g L^− 1^ acetate as the initial carbon source concentration and ammonium sulfate as the nitrogen source. After the batch phase, the pH titrant was switched from 4 M H_2_SO_4_ to glacial acetic acid to initiate pH-coupled feeding. An initial pulse of 1.5 g sodium acetate (pH 7.0) was added to start the feeding phase. Whenever the DO rose above 40%, indicating acetate depletion, additional pulses of 1.5 g sodium acetate (pH 7.0) were manually added (indicated by black arrows in Fig. 4).

**Fig. 3:**
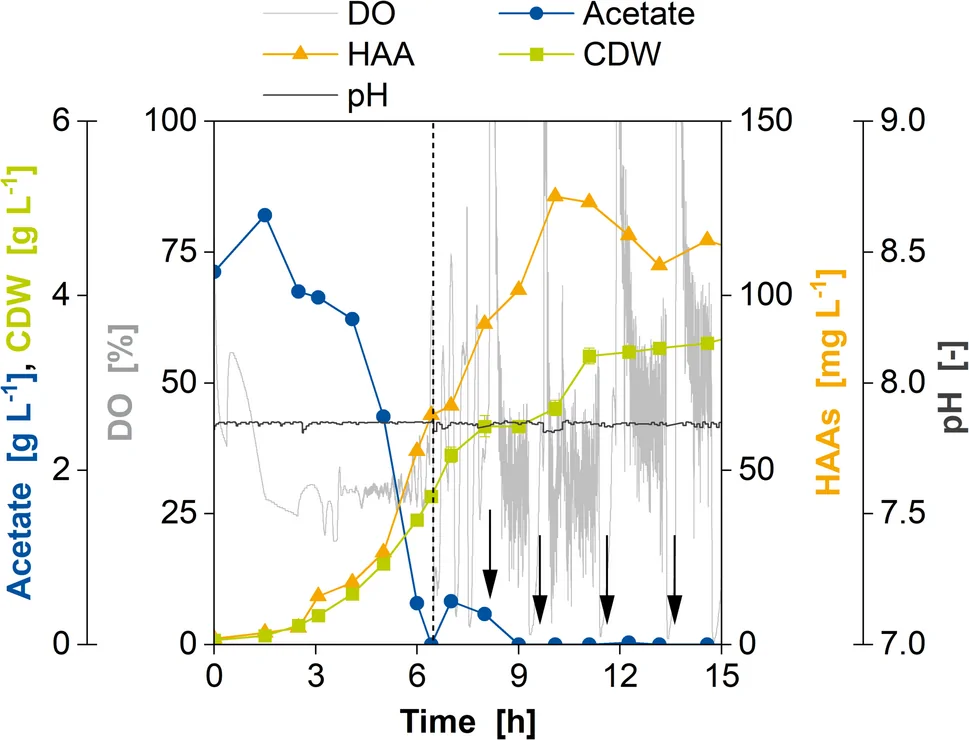
pH-coupled feeding using pure glacial acetic acid with ammonium sulfate as the nitrogen source. Membrane-aerated fed-batch cultivation using *P. putida* TALE_*rhlA*. DO (grey lines), acetate concentration (blue line, circles), HAA concentration (orange line, triangles), cell dry weight (green lines, squares), and pH (dark grey lines) over time. Cultivation conditions: MSM medium containing 5 g L^− 1^ acetate and 1x buffer, nitrogen source: ammonium sulfate, 3 L stirred-tank bioreactors with 2 L filling volume, T = 30 °C, *N* = 100–350 rpm (cascaded to maintain a DO ≥ 30% with subsequent increase of TMP to 0.3 bar and supplementation of pure oxygen (21% - 100%)), F_Air_ = 1 L min^− 1^, OD_start_ = 0.2, pH = 7.8, pH value maintained by 4 M H_2_SO_4_ (batch phase). Feed: glacial acetic acid, Pulses: 1.5 g Na-acetate (pH 7.0), CDW: cell dry weight, DO: dissolved oxygen, *n* = 1

After 6.5 h of batch cultivation, acetate was depleted, as indicated by the rising DO signal (Fig. 3). During the batch phase, a biomass concentration of 1.7 g L⁻¹ CDW, a specific growth rate of 0.61 h^− 1^, and an HAA concentration of 66 mg L⁻¹ were observed. After the first acetate pulse to initiate the feeding phase, biomass steadily increased, reaching a final concentration of 3.9 g L⁻¹ after 14 h of cultivation, but with a reduced specific growth rate of 0.02 h^− 1^.

However, acetate levels remained around 0 g L^− 1^ throughout the feeding phase, indicating carbon-limited conditions. This was further reflected in a highly oscillatory DO signal, also suggesting acetate depletion and carbon-limited conditions. Nevertheless, the pH remained close to the setpoint of 7.8 throughout the cultivation, indicating that acetate addition *via* pH-coupled feeding was sufficient to maintain pH control (Fig. 3). To compensate for the insufficient acetate supply, additional acetate pulses were manually added during the cultivation (Fig. 3, indicated by black arrows). However, despite these interventions, acetate concentrations remained low and only a small increase in biomass was observed, indicating that carbon limitation persisted throughout the feeding phase (Fig. 3). Presumably for this reason, HAA titers increased only until nine hours of cultivation and reached a maximum concentration of 130 mg L⁻¹ (Fig. 3), corresponding to a slightly enhanced Y_P/S_ of 16 mg g^− 1^, and a STY of 13 mg L^− 1^ h^− 1^ compared to the DO-based feeding strategy (Table 1). Interestingly, more carbon was used for biomass formation than in the DO-based feeding approach, reflected by a higher Y_X/S_ of 0.32 g g^− 1^ compared to the DO-based feeding strategy. As a result, the product-to-biomass yield (Y_P/X_) increased only slightly, by 11% to 47 mg g^− 1^. Despite manually supplemented acetate pulses, HAA production could not be restored, and HAA degradation was observed after 9 h, indicating that the pH-coupled feeding strategy was unable to maintain sufficient acetate availability for stable HAA production. Again, foam formation could be prevented by applying membrane aeration, avoiding the use of antifoaming agents.

A possible explanation for insufficient acetate availability is an undersupply of carbon due to the pH effects of other substances. One such candidate might be ammonium, which is added as nitrogen source. When consumed, it lowers the pH because ammonium is typically taken up as NH₃, leading to the release of free protons into the culture broth and medium acidification [50, 57]. This effect may counteract the pH increase caused by acetate metabolism. As a result, the pH would not rise enough to trigger adequate acetate feeding for continuous HAA production.

The pH-coupled fed-batch fermentation of *P. putida* TALE_*rhlA* was characterized by high biomass formation and specific growth rates, while HAA production remained comparatively low. Therefore, an alternative production strain, *P. putida* KS3 (production strain derived from the wild-type strain) [20], was compared to *P. putida* TALE_*rhlA* to identify a strain exhibiting a more favorable balance between growth and HAA production. To support strain selection for subsequent process development, the physiological characteristics of both strains were compared on 5 g L^− 1^ acetate in the microTOM System (Adolf Kühner AG, Birsfelden, Switzerland) (Additional file 2). *P. putida* TALE_*rhlA* exhibited a higher specific growth rate (0.32 ± 0.01 h^− 1^) than *P. putida* KS3 (0.27 ± 0.01 h^− 1^) and reached a higher maximum oxygen transfer rate (27.1 ± 0.8 compared to 19.9 ± 0.4 mmol L^− 1^ h^− 1^), indicating a higher respiratory activity during growth. In parallel, the TALE strain showed higher biomass formation, reaching a final CDW of 0.94 ± 0.01 g L^− 1^, whereas KS3 reached 0.62 ± 0.03 g L^− 1^ (Additional file 2). Despite its lower growth rate and biomass formation, KS3 achieved a substantially higher maximum HAA concentration (39 ± 5 mg L^− 1^) compared to the TALE strain (21 ± 2 mg L^− 1^). Consequently, the product-to-substrate yield increased 2-fold (from 4 ± 1 to 8 ± 1 mg g^− 1^). In contrast, the product-to-biomass yield increased 2.7-fold (23 ± 2 to 63 ± 7 mg g^− 1^) (Additional file 2), indicating a more favorable allocation of carbon towards HAA production in *P. putida* KS3. Similar physiological differences between both strains have also been reported in a previous study by Filbig et al. [94]. While *P. putida* KS3 also showed lower biomass formation combined with higher HAA production, higher biomass yield of the TALE strain has been attributed to the adaptive laboratory evolution (ALE) process, during which several energy-demanding cellular functions, including flagellar biosynthesis, were downregulated or inactivated, potentially increasing the energy available for biomass formation [94]. Furthermore, additional non-acetate-specific mutations identified in the evolved strain may also contribute to the altered respiratory activity and increased oxygen demand [94].

Therefore, based on these physiological characteristics, *P. putida* KS3 was selected for all subsequent bioreactor cultivations and process optimization experiments. Building on this strain selection, the following experiments aimed to further improve process performance by implementing a combined pH- and DO-coupled feeding strategy to ensure continuous carbon availability, as carbon limitation and HAA degradation were observed during pH-coupled feeding.

#### pH- and DO-coupled feeding strategy eliminates carbon limitation, but affects pH value

To overcome the carbon limitation observed during pH-coupled feeding, a feeding strategy coupled to both pH and DO was investigated using the previously selected production strain *P. putida* KS3. Since the initial pH-coupled feeding did not sufficiently prevent carbon limitation due to limited acetate supply, automated substrate addition in response to rising DO levels, indicating acetate depletion, was also implemented. In parallel, the pH of the feed solution was adjusted. Previous studies by Kövilein [82] demonstrated that increasing the pH of acetic acid-containing feed solutions can enhance carbon availability, since more feed is required to lower the pH of the cultivation broth. Therefore, the feed solution was adjusted to pH 2.0, allowing for a higher carbon input.

In the performed fermentation experiment, the batch phase started with 5 g L^− 1^ acetate. The overall cultivation time was successfully extended to 17 h, and carbon limitation was prevented, resulting in a final biomass concentration of 6.3 g L^− 1^, despite a 20% reduced specific growth rate of 0.49 h^− 1^ and a 32% reduced Y_X/S_ of 0.22 g g^− 1^ compared to the pH-coupled feeding strategy (Additional file 3). Throughout the cultivation, HAA production remained growth-coupled, indicating activity of sigma70 factor-dependent promoters controlling *rhlA* expression [21] (Additional file 3). Consequently, the final HAA concentration increased 4.2-fold to 545 mg L⁻¹, resulting in a 2.4-fold enhanced STY of 31 mg L^− 1^ h^− 1^ compared to the pH-coupled feeding strategy with the TALE strain (Additional file 3). Although the product-to-substrate yield increased only 1.2-fold to 19 mg g^− 1^ due to the 3.5-fold higher acetate supply (57 g versus 16 g acetate; Table 1), the product-to-biomass yield increased 1.8-fold, indicating a more efficient conversion of biomass into HAA (Table 1). These results demonstrate that the combined pH- and DO-coupled feeding strategy successfully prevented carbon limitation and further enhanced process performance when applied to the selected production strain *P. putida* KS3. However, the approach led to pronounced pH fluctuations throughout the cultivation (Additional file 3), motivating further optimization of substrate feeding. Therefore, urea was investigated as an alternative nitrogen source, as its pH-neutral metabolism was expected to improve pH-controlled substrate feeding.

### Urea as nitrogen source for more stable pH-coupled feeding strategy

To improve the sustainability and process performance of HAA production with *P. putida*, urea was investigated as an alternative nitrogen source. Urea is expected to enhance the overall sustainability of the fermentation process due to its potential for sustainable production from renewable energy, atmospheric nitrogen, and captured CO_2_ [98, 99]. Furthermore, the use of urea can reduce salt accumulation in the cultivation broth compared to ammonium sulfate [100]. From a process perspective, the pH-neutral metabolization of urea is of interest, as it avoids the acidification associated with ammonium uptake and thereby enables a more direct coupling between substrate consumption and the resulting pH increase. Consequently, pH-coupled feeding strategies can be facilitated, potentially simplifying process control [101, 102].

#### Urea promotes comparable growth and HAA production in shake flasks

As a first step, the suitability of urea as nitrogen source was evaluated in a shake flask experiment comparing urea- and ammonium-based growth (same C/N ratio of 5) and HAA production in *P. putida* KS3, with end-point determination of CDW, HAA concentration, and pH. Furthermore, growth was monitored using an online monitoring device for non-invasive measurement of the OTR.

**Fig. 4:**
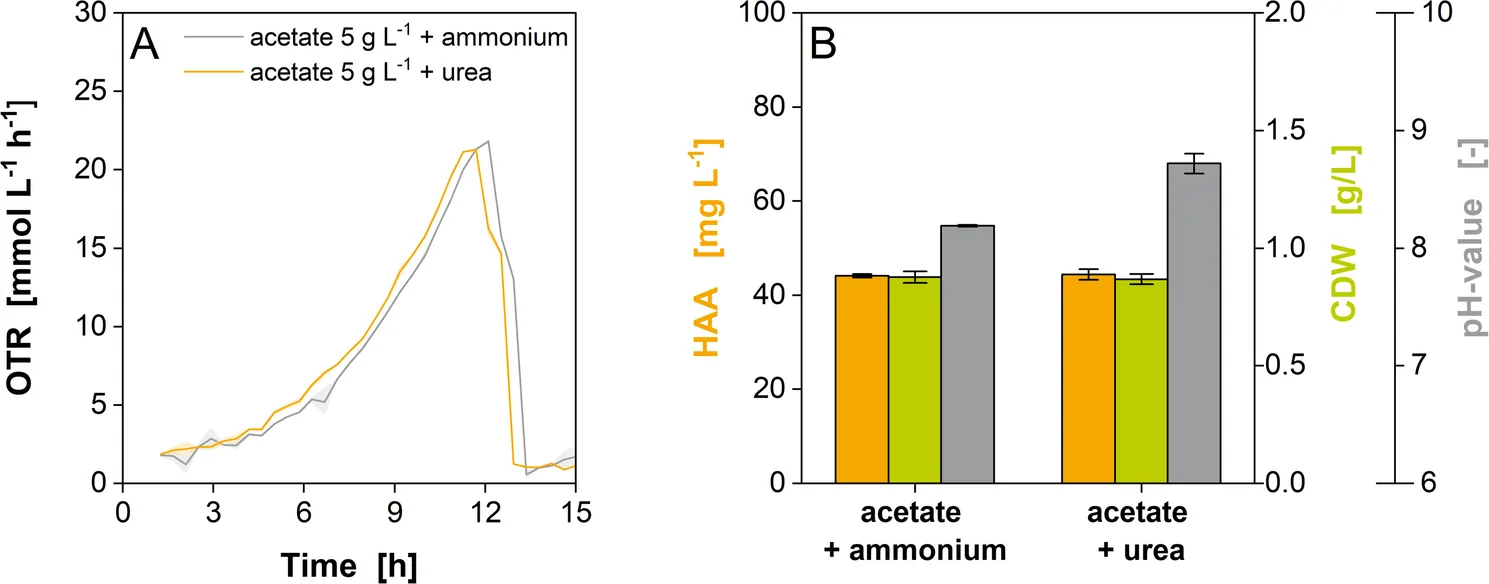
Investigating urea as nitrogen source in shake flask cultivation with *P. putida* KS3. **A** OTR profiles over time, **B** Comparison of HAA concentrations (orange), biomass (green), and final pH value (grey) at the end of the cultivation (as soon as OTR drops, indicating substrate depletion). Cultivation conditions: MSM medium containing 5 g L^− 1^ acetate, either 2 g L ^1^ammonium sulfate or 0.91 g L^− 1^ urea (C/N ratio: 5), and 4x buffer, T = 30 °C, pH_start_ = 7.0, pH_end_ = 8.0 and 8.5, OD_start_ = 0.1; V_fill_ = 25 mL, V_Shake flask_ = 500 mL, *N* = 300 rpm, d = 50 mm, *n* = 3 (offline samples for end point determination), Measurement of OTR (n =2): TOM System. The experiment was conducted in duplicates (*n* = 2)

The results of the cultivation experiment comparing urea- and ammonium-based media revealed fully comparable growth and biomass formation (Fig. 4). In detail, both OTR profiles followed the same exponential growth pattern, resulting in a maximal OTR of 22 ± 1 mmol L^− 1^ h^− 1^ after 11.3 and 11.7 h of cultivation, corresponding to a specific growth rate of 0.36 h^− 1^ (calculated from OTR during exponential growth phase until 11.3 and 11.7 h of cultivation) for both ammonium and urea-based cultivation, resulting in 0.9 ± 0.1 g L^− 1^ of CDW at the end of the cultivation (Fig. 4A, B). Besides growth, HAA production was comparable at 44 ± 1 mg L^− 1^ in both cultivations (Fig. 4B). As expected, the final pH in the urea-based cultivation was higher than in the ammonium reference (8.7 ± 0.1 versus 8.2 ± 0.1), indicating that the pH value is mainly influenced by acetate uptake since urea is metabolized without any influence on the pH value (Fig. 4B) [101, 102]. This effect could be beneficial for fed-batch processes with pH-coupled feeding, as it could potentially prevent carbon limitation by counteracting pH decreases caused by ammonium metabolization.

#### Urea facilitates stable pH-coupled feeding and enhances HAA production

Based on these results, urea was subsequently evaluated as nitrogen source in pH-coupled fed-batch fermentations to investigate its influence on process control and carbon limitation. For initial testing and to avoid complications from excessive foaming during cultivation, the non-producing *P. putida* wild-type strain was used to investigate the general performance under urea-based feeding conditions (Additional file 4). Therefore, a bubble-aerated fermentation was performed with 5 g L^− 1^ acetate. Again, the pH of the feed solution was adjusted to 2.0. In contrast to the other fermentation experiments, the feeding solution served as the pH titrant from the beginning of cultivation and contained urea to prevent nitrogen limitation during fermentation. Therefore, no distinct batch phase occurred. The experiment demonstrated that stable feeding control could be achieved with urea as nitrogen source. Within a cultivation time of 13 h, the acetate concentrations ranged between 5 g L^− 1^ in the beginning and 2 g L^− 1^, thereby preventing any carbon limitation (Additional file 4). At the end of the cultivation, a final biomass concentration of 6.6 g L^− 1^ with a specific growth rate of 0.31 h^− 1^ was achieved. In total, 27 g of acetate were fed in the reactor, corresponding to a biomass-to-substrate yield of 0.24 g g^− 1^ (Additional file 4). These results confirm the suitability of the feeding strategy for further process development with the HAA-producing strain, which was tested in the subsequent fermentations.

To investigate the applicability of urea as nitrogen source under production conditions, the pH-coupled feeding strategy was tested for HAA synthesis using the HAA-producing *P. putida* KS3 strain. The fermentation started with an initial acetate concentration of 5 g L^− 1^; to avoid foaming, membrane aeration was again applied.

**Fig. 5:**
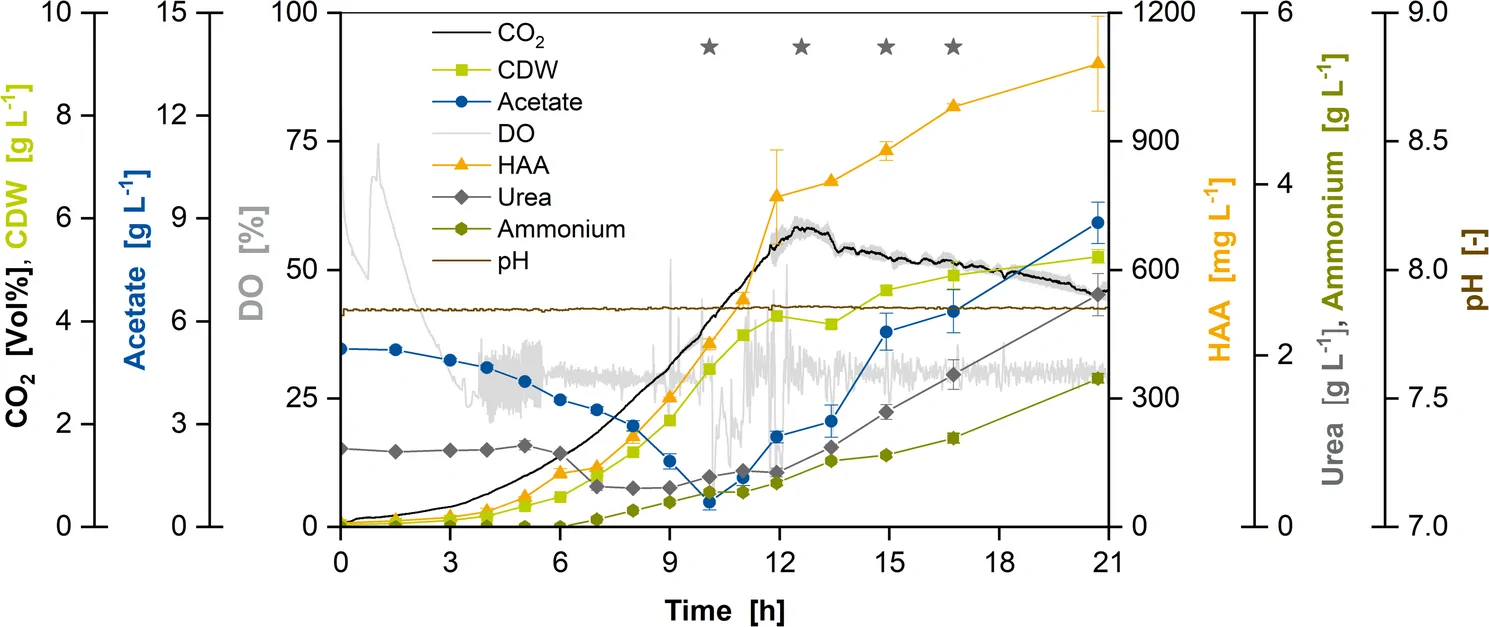
Optimized pH-coupled feeding strategy with urea as nitrogen source using *P. putida* KS3. Membrane-aerated fed-batch cultivation with offgas CO_2_ signals (black line), CDW (green line, squares), acetate concentration (blue line, circles), DO (light grey lines), HAA concentration (orange line, triangles), urea concentration (grey, diamonds), ammonium concentrations (dark green, hexagons), and pH (dark grey lines) over time. Cultivation conditions: MSM medium containing 5 g L^− 1^ acetate and 1x buffer, nitrogen source: urea, 3 L stirred-tank bioreactors with 2 L filling volume, T = 30 °C, *N* = 100–350 rpm (cascaded to maintain a DO ≥ 30% with subsequent increase of TMP to 0.3 bar and supplementation of pure oxygen (21%-100%)), F_Air_ = 1 L min^− 1^, OD_start_ = 0.2, pH = 7.8, pH value maintained with feed solution containing acetic acid and urea with a pH value of 2.0, stars indicate the addition of trace elements, CDW: cell dry weight, DO: dissolved-oxygen. Fermentation was performed in duplicates (*n* = 2). Error bars represent the standard deviation of the mean

Applying the urea-based pH-coupled feeding strategy, the total cultivation time could be extended to 21 h without HAA degradation. During the first twelve hours of cultivation, exponential growth could be observed with a specific growth rate of 0.45 ± 0.02 h^− 1^, also reflected by an exponential increase of the CO₂ signal of up to 5.8 ± 0.1 vol% (Fig. 5). In addition, the pH remained close to the setpoint of 7.8 throughout the entire cultivation, indicating stable process control under urea-based pH-coupled feeding conditions (Fig. 5). The specific growth rate was comparable to that achieved in the pH- and DO-coupled feeding experiment. After twelve hours, CO_2_ levels declined until the end of cultivation, accompanied by reduced biomass formation and slower HAA production (Fig. 5). During this period, additional medium components were added to the reactor to address a possible second-substrate limitation and restore growth (indicated by stars); however, this did not affect growth or CO_2_ levels. In general, HAA production remained growth-coupled throughout the entire fermentation as it clearly correlated with biomass formation (Fig. 5). The acetate profile differed from that observed in the pH-coupled fermentation of the non-producing strain: substrate levels continuously decreased until around ten hours and began to accumulate shortly before the CO₂ signal dropped until the end of cultivation to a final value of 8.9 ± 0.6 g L^− 1^. Urea concentration followed a similar trend, increasing after ten hours of cultivation, and reaching a final concentration of 2.7 ± 0.2 g L^− 1^ (Fig. 5). Urea accumulation was likely caused by increasing ammonium concentrations during the final cultivation phase, which are known to inhibit urea uptake [101]. In this phase, the previously self-regulating, growth-coupled acetate uptake was apparently no longer maintained, leading to substrate accumulation as biomass formation slowed down. Nevertheless, no carbon limitation occurred throughout the cultivation, allowing continuous HAA production (Fig. 5). The process yielded the highest titer in this study, reaching 1,080 ± 115 mg L^− 1^ at the end of cultivation, corresponding to a 1.6-fold higher STY of 51 ± 5 mg L⁻¹ h^− 1^ compared to the pH- and DO-coupled feeding strategy (Table 1).

However, several challenges became apparent during the experiment. Exponential, unlimited growth stopped after approximately twelve hours, leading to acetate accumulation in the medium. Several pulses containing medium components had no positive effect on growth (as indicated with stars, Fig. 5), indicating the need to identify possible growth-limiting factors to potentially prolong the exponential growth phase and maintain high production rates. In contrast to the pH-coupled fermentation with the non-producing strain, in this experiment, acetate levels continuously decreased during the exponential growth phase and increased as soon as growth slowed, even though urea, a pH-neutral nitrogen source rather than ammonium, was used. This might be attributed to other pH-active molecules in the culture medium. The produced HAAs, with their acidic carboxyl groups, are pH-active molecules produced in significant amounts during cultivation. Rhamnolipids are reported to exhibit a pK_a_ of around 5.5 [103, 104]. Since HAAs and rhamnolipids contain the same terminal carboxyl group and therefore exhibit comparable acid-base behavior, the pK_a_ of HAAs is assumed to be in a similar range. This suggests that HAAs also contribute to a decrease in pH, as the molecules are mainly deprotonated at the cultivation pH of 7.8, especially toward the end of cultivation.

Compared to the pH- and DO-coupled feeding strategy, acetate utilization and HAA production were significantly more efficient in this experiment. Almost 20% less acetate was fed into the reactor (47 g versus 57 g), yet a doubled final HAA concentration was achieved. Biomass yield remained comparable (0.23 ± 0.01 g g^− 1^), suggesting improved carbon utilization toward product formation rather than growth. This effect is also reflected in the yields: both the product-to-substrate yield (48 ± 4 mg g^− 1^) and the product-to-biomass yield (205 ± 17 mg g^− 1^) are more than doubled compared to the pH- and DO-coupled fermentation experiment (Table 1). Urea thereby contributed to an overall improved process performance. In contrast to the pH- and DO-coupled feeding strategy, which caused strong pH fluctuations, urea feeding enabled stable pH throughout the cultivation, resulting in more efficient product formation and substrate utilization. Moreover, because only a single feed solution was used in this fermentation process, control was simplified. Besides these process-related benefits, physiological effects caused by urea uptake may have also contributed to higher productivity: the intracellular cleavage of urea into CO_2_ and ammonium could have increased the intracellular CO_2_ concentrations, thereby promoting metabolic flux towards fatty acid *de novo* synthesis, which is the precursor pathway for HAA formation [14, 16]. Moreover, urea has previously been shown to increase expression levels of genes regulated by sigma70 promoters, potentially as a stress response to elevated urea concentrations [105–107]. However, in this study, the urea concentration was relatively low compared to those used in previous studies.

In conclusion, the pH-coupled feeding strategy with urea as nitrogen source was successfully implemented, yielding stable, continuous HAA production without degradation. Regarding the overall sustainability of the process, urea not only stabilizes the bioprocess but also offers ecological benefits, especially when synthesized from renewable energy, nitrogen, and CO₂, both captured from air, making it an attractive nitrogen source for sustainable bioprocesses [98]. To further enhance sustainability, CO₂-derived substrates such as chemocatalytically produced acetate and acetate-formate mixtures were evaluated to reduce potentially the overall carbon footprint of the process.

### Implementing CO_2_-derived acetate and acetate-formate mixtures for HAA production

In the context of developing sustainable bioprocesses, the use of CO_2_-derived carbon sources is a promising strategy to reduce dependence on feedstocks burdened with the food-vs-fuel dilemma. To further improve the overall sustainability of the bioprocess, different CO_2_-derived substrates were evaluated. First, acetate generated *via* chemocatalytic isomerization of methyl formate was tested (substrate mixture 1, Table 2) [47]. Second, the protocol described by Guntermann [84] was optimized, which utilizes a biphasic system with a ruthenium catalyst for hydrogenation of CO_2_ to formate with NaOH as stabilizing base for the reaction. In this study, sodium acetate was used as the base, resulting in an acetate-formate mixture with a ratio of 5:1 (w/w, substrate mixture 2, Table 2). Finally, both approaches described above were combined: the acetate obtained by the method described by Jürling-Will [47] was purified and used as stabilizing base for the hydrogenation of CO_2_ to formate, resulting in a complete CO_2_-derived acetate-formate mixture (substrate mixture 3, Table 2). All three substrate mixtures were evaluated for biocompatibility, growth, and HAA production. In the following experiments, the strain *P. putida* TALE_*rhlA* was used due to its improved tolerance towards acetate and derivatives.

**Table 2:** Characteristics of CO_2_-derived substrate mixtures used in this work

Products	Substrate mixture 1	Substrate mixture 2	Substrate mixture 3
	CO_2_-derived acetic acid mixture	CO_2_-derived acetate-formate mixture	Complete CO_2_-derived acetate-formate mixture
Educts	methyl formate (potentially derived from CO_2_)	CO_2_ and sodium acetate (obtained from Sigma-Aldrich)	CO_2_ and sodium acetate (purified from mixture 1)
Catalytic reaction	Isomerization of methyl formate (derived from CO_2_)	Hydrogenation of CO_2_ with sodium acetate as stabilizing base	Hydrogenation of CO_2_ with sodium acetate as stabilizing base
Catalyst metal	Palladium	Ruthenium	Ruthenium

#### Unpurified acetic acid mixtures from Pd-catalyzed isomerization of methylformate not compatible with *P. putida*

The first growth experiment with 5 g L^− 1^ of the chemically derived acetic acid mixture 1 as sole carbon source resulted in no detectable growth (Additional file 5). To identify potential inhibitory compounds, the main side components that occurred during the reaction, methyl acetate, ethyl acetate, formic acid, and methanol [47], were subsequently tested in combination with 5 g L^− 1^ sodium acetate. Using a mixture containing the side components and acetate, growth was observed, but with a reduced specific growth rate of 0.34 h⁻¹ (compared to 0.43 h^− 1^ for the sodium acetate reference), indicating mild inhibition (Additional file 5). Subsequent biocompatibility tests of the individual components in the acetate mixture revealed that residues of the palladium catalyst and associated components, particularly the phosphine ligands and potential degradation products such as phosphine oxides, strongly influenced cell growth (Additional file 5). This indicates that the presence of catalyst (components) even in small amounts in the product mixture is toxic to cells [108, 109]. and a purification step to remove the catalyst is needed to ensure biocompatibility.

#### CO_2_-derived acetate-formate mixtures are biocompatible

**Fig. 6:**
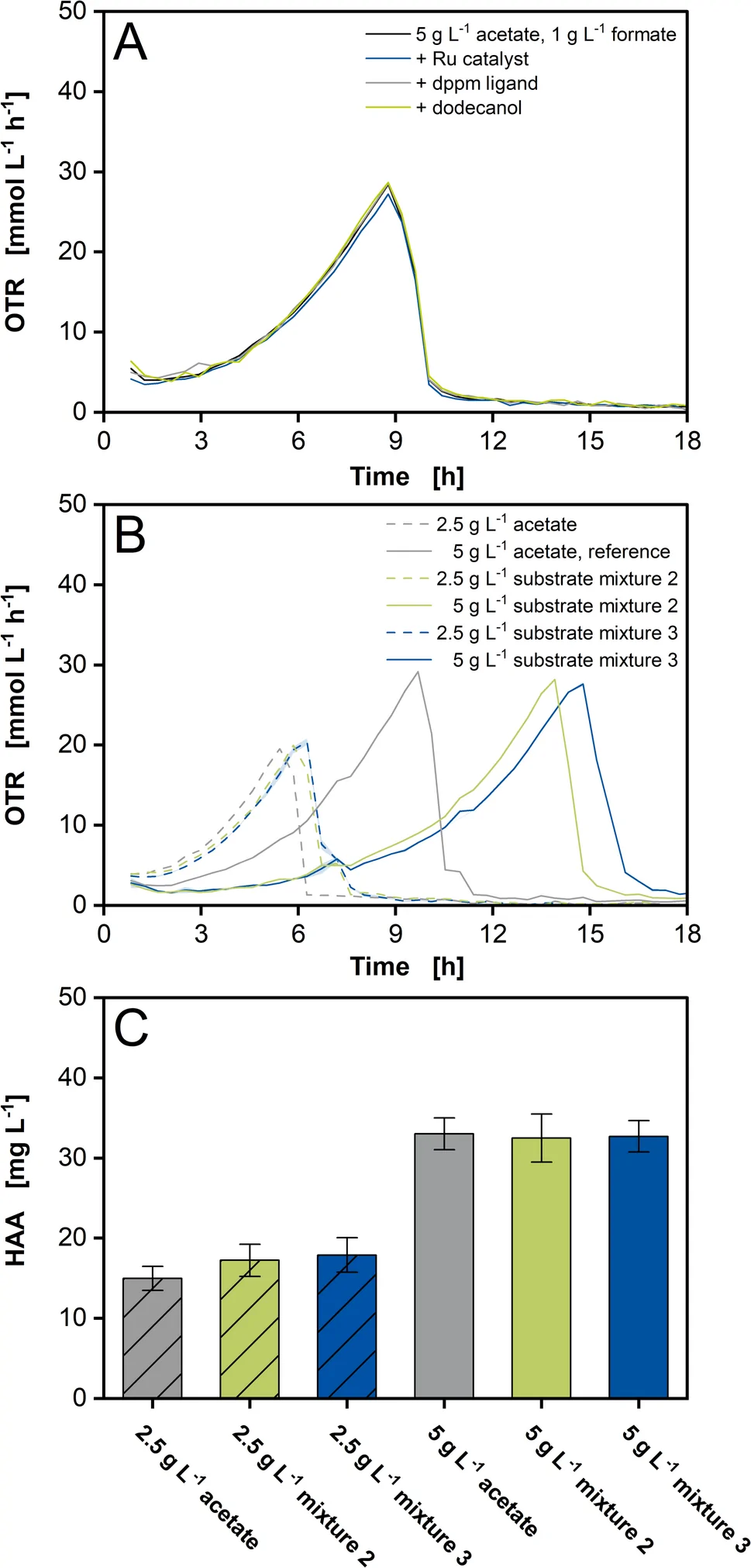
Investigation of different CO_*2*_-derived substrate mixtures of *P. putida* TALE_*rhlA* in shake flasks. Biocompatibility test (A), growth (B), and HAA production (C) on CO_2_-derived acetate-formate mixtures 2 and 3 compared to acetate reference. To test the biocompatibility, solvent as well as catalyst compounds were added in individual cultivations with 5 g L^− 1^ sodium acetate and 1 g L^− 1^ sodium formate (ratio 5:1). For the growth and production experiment, different concentrations (2.5 g L^− 1^ (dashed lines, filled columns) and 5 g L^− 1^ (continuous line, plain columns) of the substrates were investigated, respectively). In comparison to the acetate reference (grey), the CO_2_-derived formate-acetate mixture 2 (green) and the completely CO_2_-derived formate-acetate mixture 3 (blue) were tested. MSM Medium, T = 30 °C, pH_start_ = 7.0, OD_start_ = 0.1, V_L_ = 25 mL in 500 mL shake flasks, *n* = 300 rpm, *n* = 2 (OTR monitoring), *n* = 3 (HAA production); Measurement of OTR: TOM System. Error bars represent the standard deviation of the mean

To overcome the observed inhibitory effects associated with palladium-catalyzed acetate synthesis, an alternative production route was utilized. This approach uses a ruthenium catalyst for the hydrogenation of CO_2_ in the presence of sodium acetate in a biphasic system with dodecanol acting as catalyst phase and water as the product phase, ensuring an intrinsic catalyst separation. This catalytic system yielded an acetate-formate mixture with a ratio of 5:1 (w/w). As a first step, the biocompatibility of individual side components, such as the organic solvent and catalyst-associated compounds, was evaluated (Fig. 6A). For this purpose, each component was added at its respective concentration present in the original mixture to a cultivation medium containing 5 g L⁻¹ sodium acetate and 1 g L⁻¹ sodium formate, to maintain a constant substrate ratio. The results demonstrated full biocompatibility of all tested compounds of the mixture, with growth behavior comparable to the reference cultivation and specific growth rates of 0.34 h⁻¹ and a maximum OTR of 29 mmol L^− 1^(Fig. 6A).

An acetate-formate mixture derived entirely from CO_2_ was obtained combining the two approaches. First, the acetate mixture obtained from the palladium-catalyzed methylformate isomerization was purified by distillation and treated with sodium hydroxide. The resulting sodium acetate was then used as a base in the hydrogenation of CO_2_ to formate, yielding an acetate-formate mixture, which is now formally entirely derived from CO_2_ (substrate mixture 3, Table 2). In a subsequent experiment, the growth and HAA production of *P. putida* TALE_*rhlA* was investigated using two different concentrations (2.5 g L⁻¹ and 5 g L⁻¹) of the acetate-formate mixture 2 and the completely CO_2_-derived substrate mixture 3 compared to corresponding reference cultivations with sodium acetate and sodium formate (2.5 g L⁻¹ + 0.5 g L^− 1^ and 5 g L⁻¹ + 1 g L^− 1^) (Fig. 6B). At the lower concentration of 2.5 g L⁻¹, OTRs showed profiles similar to the reference, with comparable specific growth rates of 0.53 h⁻¹, 0.51 h^−^¹, and 0.53 h⁻¹. However, at the higher concentration (5 g L⁻¹), both cultivations with substrate mixes 2 and 3 showed slower growth with prolonged lag phases and lower specific growth rates compared to the reference cultivation, suggesting a concentration-dependent inhibitory effect of both mixtures (Fig. 6B). Regarding HAA production, product formation was fully comparable to the reference experiments, reaching approximately 17 ± 2 mg L⁻¹ at 2.5 g L⁻¹ and 33 ± 2 mg L⁻¹ at 5 g L⁻¹ substrate mixture (Fig. 6C).

In summary, palladium-catalyzed isomerization of methyl formate can be combined with biphasic ruthenium-catalyzed CO_2_ hydrogenation to produce a bio-compatible formate-acetate feed. Although a purification step is necessary after the first process, the aqueous phase after the hydrogenation step can be used directly in the bioprocess. Interestingly, as shown in literature, formate could serve as a beneficial co-substrate, providing additional energy and supporting growth [32, 110]. However, this beneficial property could not be demonstrated in this study, and further investigation is needed. To further evaluate the potential of formate as an HAA-promoting substrate, continuous cultivations could be performed [32].

## Conclusion

In conclusion, the results of the study demonstrate a dual strategy for sustainable HAA production: optimizing the fermentation process by applying a urea-based fed-batch strategy to enhance process performance and integrating CO_2_-derived substrates as renewable carbon sources. The here shown interdisciplinary chemo- and biocatalytic approach to use CO_2_ as carbon source opens up landfree biotechnology, in which very limited resources of land use are required, when compared to corn, wheat and sugar beat farming. The study presents a promising approach toward fully sustainable HAA production, enhancing the role of acetate as next-generation feedstock in an emerging bioeconomy. Addressing HAA degradation pathways *via* genetic engineering to improve HAA synthesis and fully understanding formate metabolism will be key to further enhancing productivity and overall process performance.

## Materials and methods

### Bacterial strains and medium

Cultivation experiments were carried out using *Pseudomonas putida* KT2440 harboring genomically integrated *rhlA* gene from *P. aeruginosa* PAO1, resulting in the strain *P. putida* KT2440 *att*Tn7::P_ffg_-*rhlA*, referred to as *P. putida* KS3 in this study. The HAA producing strain was constructed and characterized in a previous study [20], based on *P. putida* KT2440 [111]. To enhance the tolerance towards acetate, an ALE experiment for enhanced tolerance towards the substrate was performed (TALE) using an automated liquid handler platform [92, 93]. This strain was constructed and characterized in a previous study [94], subsequently equipped with the HAA production gene, resulting in the strain *P. putida* KT2440 TALE *att*Tn7::P_ffg_-*rhlA*, referred to as *P. putida* TALE *rhlA* in this study [94]. A detailed description of the adaptive laboratory evolution procedure, genomic characterization, and investigations of the identified mutations were performed in Filbig et al. [94] This strain was used to investigate different CO_2_-derived acetate and formate mixtures and for the first fermentation experiments.

For the initial preculture, all strains were cultivated in LB medium overnight containing (per L) 5 g yeast extract, 10 g tryptone, 5 g NaCl, supplemented with 10 g glucose, and 25 mg gentamycin to select for producing colonies. A mineral salts medium (MSM) adapted from Hartmans [112] was applied for all cultivation experiments and second and third precultures and contained (per L) 5 g acetate or 10 g glucose, a phosphate buffer consisting of 15.52 g K_2_HPO_4_, 6.52 g NaH_2_PO_4_ and either and 2 g (NH_4_)_2_SO_4_ or 0.91 g CO(NH_2_)_2_ as nitrogen source. Additionally, it contained the trace elements 10 mg EDTA, 0.1 mg MgCl_2_ · 6 H_2_O, 2 mg ZnSO_4_ · 7 H_2_O, 1 mg CaCl_2_ · 2 H_2_O, 5 mg FeSO_4_ · 7 H_2_O, 0.2 mg Na_2_MoO_4_ · 2 H_2_O, 0.2 mg CuSO_4_ · 5 H_2_O, 0.4 mg CoCl_2_ · 6 H_2_O, and 1 mg MnCl_2_ · 2 H_2_O. Since the pH value could be controlled by acid or base in bioreactor cultivations, the phosphate buffer concentration was lowered to a quarter of its initial concentration. For DO-based feeding, a concentrated MSM feeding solution containing (per L) 200 g acetate, 7.76 g K_2_HPO_4_, 3.26 g NaH_2_PO_4_, 40 g (NH_4_)_2_SO_4_, and a 3.3-fold concentrated trace element solution, also modified from Hartmans [112], was used.

### Cultivation conditions

#### Preculture

For all cultivations, the preculture protocol was carried out as follows: first, cryopreserved cultures of the production strain were streaked out on an LB agar plate with gentamycin and incubated overnight at 30 °C. Fresh cell material was transferred into 100 mL shake flasks, containing 10 mL of LB medium with 10 g L^− 1^ glucose, which was incubated at 30 °C, with a shaking frequency of 300 rpm (shaking diameter of 50 mm). For the second preculture, MSM medium containing 10 g L^− 1^ glucose was inoculated to OD_600_ = 0.1 (shake flasks, 10% filling volume, 300 rpm, 50 mm shaking diameter, 30 °C) and cultivated until the early exponential phase. Afterward, a third preculture containing MSM and 5 g L^− 1^ acetate was inoculated (shake flask, 10% filling volume, 300 rpm, 50 mm shaking diameter, 30 °C). Cells were harvested in the mid-exponential phase (OD_600_ 2-2.5) to inoculate production experiments in shake flasks or bioreactors.

#### Cultivations with online monitoring of oxygen and carbon dioxide transfer rates

Different production strains were compared in microtiterplates (MTPs) using a device for online monitoring of oxygen transfer rates (OTR), the microTOM (transfer rate online monitoring) device (Adolf Kühner AG, Birsfelden, Switzerland). For OTR measurements, 96-square deep-well MTPs (HJ-Bioanalytik GmbH, Erkelenz, Germany) were utilized. The MTPs were sealed with a sterile gas-permeable membrane (Breathe Easier, Z763624, Sigma-Aldrich). MTPs were shaken at 300 rpm with a 50 mm shaking diameter at 30 °C. MicroTOM cultivations were inoculated to an OD_600_ of 0.1 in MSM medium containing 5 g L^− 1^ acetate and performed in quadruplicates.

Investigations of nitrogen sources and CO_2_-derived substrate mixtures were performed using the transfer rate online measurement (TOM) device for shake flasks (Adolf Kühner AG, Birsfelden, Switzerland), an online monitoring device for non-invasive measurement of the oxygen (OTR) and carbon dioxide transfer rates (CTR). For TOM cultivations, 500 mL shake flasks with a filling volume of 5% were inoculated to OD_600_ = 0.1 containing MSM medium with 5 g L^− 1^ acetate. The cultivations were performed at 30 °C and 300 rpm (50 mm shaking diameter) in duplicates.

### Bioreactor cultivations

All fermentations were performed in the BioFlo120 bioreactor system under the control of the DASware control software package Version 6.4.0 (both Eppendorf AG, Hamburg, Germany). The bioreactors were equipped with integrated control systems for pH and temperature. The cultivation temperature was set to 30 °C, and the pH was adjusted to the desired value (7.0–7.8) *via* the addition of acid and base (4 M H_2_SO_4_ and 4 M KOH), monitored with online pH sensors (EasyFerm Plus PHI K8 225, Hamilton, Rena, USA). The dissolved oxygen (DO) was measured with an online DO sensor (InPro 6800, Mettler-Toledo, Columbus, Ohio, USA). Exhaust gas with O_2_ and CO_2_ concentrations was monitored by BlueVary Sensors and BueVis software v4.6.5 (BlueSens gas sensor GmbH, Herten, Germany). All bioreactor cultivations were inoculated to an initial OD_600_ of 0.2. Batch phases were conducted with either 5 g L^− 1^ or 2.5 g L^− 1^ acetate.

#### Surface-aerated batch fermentations

All surface-aerated batch fermentations were conducted in a 1.3 L stirred-tank bioreactor with a working volume of 0.5 L. The agitation shaft was equipped with two six-blade Rushton turbines (diameter = 5.3 cm). The airflow was set to a constant 0.5 L min^− 1^. The DO level was maintained above 30% by cascaded agitation (400–800 rpm) to prevent oxygen limitation. When foaming occurred, 0.1 mL of sterile antifoam 204 (Sigma Aldrich, Darmstadt, Germany) was added through the septum manually.

#### Bubble-free membrane-aerated fermentation setup

All fed-batch fermentation experiments were performed at a pH value of 7.8 using 3 L glass bioreactors with 2 L working volume and were equipped with the MemStir (Biothrust GmbH, Aachen, Germany) [83], serving both as an agitation and bubble-free aeration system. The MemStir was installed in the bioreactor instead of the agitation shaft and sparger. The airflow was set to a constant 1 L min^− 1^ and DO levels were maintained above 30% by first cascaded agitation (100–350 rpm), then an increase of the transmembrane pressure to 0.3 bar and subsequent oxygen supplementation (21%−100%) to prevent oxygen limitation. No foam sensor or antifoaming agent supply was applied in this reactor setup. The whole bioreactor was autoclaved before fermentation (without water), and sensors were installed after disinfection with 70% ethanol.

#### Feeding strategies

DO-based carbon-limiting feeding was investigated as described in previous studies [52, 75, 83]. DO-based feeding started after acetate depletion, as indicated by DO rising above 70%, triggering the feed pump and consequently decreasing DO. The feed flow rate was set constant to 43 mL h^− 1^ and stopped when a DO of 40% was reached. As soon as acetate was depleted again, as indicated by a rise in the DO signal to 70%, the next feeding cycle was started. During the feeding phase, the amount of supplemented oxygen was further increased manually if the DO fell below 15%. When OD_600_ reached values of 10 and 20, additional trace element solution, phosphate, and nitrogen source were pulsed manually through the septum.

Fed-batch processes with pH-coupled feeding as sole feeding strategy were conducted using glacial acetic acid as a sole feed solution, both serving as pH titrant and carbon source. Acetic acid was automatically pumped into the reactor every time the pH rose above 7.8. Using ammonium as a nitrogen source, the batch phase was conducted with 4 M H_2_SO_4_ as pH-adjusting agent, while for urea-based surfactant production, acetic acid was used as pH titrant over the whole cultivation time. Every time the DO rose over 40%, indicating acetate depletion, concentrated pH-neutral acetate feed solution was automatically pulsed into the reactor with a volume of 10 mL feed solution per feed shot.

Fed-batch fermentation experiments with DO- and pH-coupled feeding of acetic acid were performed with acetic acid coupled to both feeding pumps. Feed pumps were activated either to maintain a pH of 7.8 or if the DO increased by 10% to 40% DO. During the batch phase, pH was maintained *via* 4 M H_2_SO_4_ as pH-adjusting agent.

### Analytical methods

#### Analysis of bacterial growth

Bacterial growth was determined by measuring the optical density at 600 nm (OD_600_) using an Ultrospec 10 cell density meter (Biochrom, Cambridge, United Kingdom) with 0.9% NaCl as blank and for sample dilution. For the production host with integrated production genes (*P. putida* KS3), the conversion factor between OD_600_ and cell dry weight (CDW) was determined to be 0.263 g L^− 1^ CDW per unit OD_600_. For the production strain with enhanced tolerance towards acetate used in this work (TALE_*rhlA*), a CDW-OD_600_ conversion factor of 0.298 g L^− 1^ CDW per unit OD_600_ was determined.

#### Quantification of acetate *via* HPLC-UV

Acetate concentrations were measured using a Dionex UltiMate 3000 system (Thermo Fisher Scientific, Waltham, USA) containing the column compartment TCC-3000SD, the autosampler WPS-3000TSL, and the isocratic pump module ISO-3100SD connected to the UV-detector VWD-3100 (all Thermo Fisher Scientific, Waltham, MA, USA) with a Metab-AAC ion exchange column (ISERA GmbH, Düren, Germany). As solvent for isocratic elution 5 mM H_2_SO_4_ was used with a flow rate of 0.6 mL min^− 1^ at 40 °C. A volume of 10 µL was injected for analysis, and peak detection was performed with a UV detector at 210 nm and completed after 19 min. Prior to measurement, the supernatant of the centrifuged sample (13,000 rpm, 3 min) was filtered through a 0.22 μm filter with a cellulose-acetate membrane (EXCALIBUR, Kitchener, Ontario, Canada). If more than 1 mL of antifoam was applied during fermentation, samples were additionally diluted 1:10 with filtered ultrapure water.

#### Quantification of HAAs *via* HPLC-CAD

For the determination of HAA concentrations, analytical methods and sample preparations were performed according to Filbig [14]. Briefly, samples from cultivation experiments were mixed 1:1 (v/v) with acetonitrile, stored overnight at 4 °C, then centrifuged at 13,000 rpm for 2 min and filtered through Phenex RC syringe filters (0.2 μm, Phenomenex, Torrance, CA, USA).

For separation of HAA congeres, a Dionex UltiMate 3000 system (Thermo Fisher Scientific, Waltham, MA, USA) consisting of the column compartment TCC-3000SD, the autosampler WPS-3000TSL, and a dual gradient pump connected to a Corona Veo Charged Aerosol Detector (CAD) (all Thermo Fisher Scientific, Waltham, MA, USA) was used. Separation of the glycolipid congeners took place using a NUCLEODUR C18 Gravity column (150 × 3 mm, particle size 3 μm; Macherey-Nagel GmbH & Co.KG, Düren, Germany). The method lasted for 11 min, and the column oven was heated to 40 °C. The mobile phase consisted of two elution buffers: 0.2% v/v formic acid in acetonitrile (A, vol/vol) and 0.2% v/v formic acid in ultrapure water (B, vol/vol). The injection volume was set to 3 µL. Elution was performed using an analytical and inverse gradient, both with a flow rate of 0.425 mL min^− 1^. An analytical gradient was applied, starting with a ratio of 70% A and 30% B. After 0.5 min, the share of A was increased linearly to 100% until minute 4.5 and kept constant for 1 min. Within the next 30 s, the ratio decreased to the starting conditions of 70% A and 30% B and was kept constant until the end of the measurement. An inverse gradient was applied to maintain a constant mobile-phase composition at the CAD detector, as calculated by the software in the “keep solvent composition” mode (Chromeleon 7.2.10). For establishing the inverse gradient, an offset was determined. As a standard, a triple standard consisting of tetradecyl-β-D-maltoside, deoxycholic acid, and 1-monoolein was used.

#### Quantification of urea *via* HPLC-UV

For the measurement of urea concentrations, 0.5 mL of the samples was diluted with 0.5 mL of 100% acetonitrile and incubated overnight at 4 °C to induce protein precipitation. After incubation, samples were centrifuged (13,000 rpm, 5 min) and sterile-filtered with solvent-resistant syringe filters (Phenex RC syringe filters, 0.2 μm, d = 4 mm, Phenomenex, Torrance, USA). The measurement was conducted using a Dionex UltiMate 3000 HPLC system containing the column compartment TCC-3000SD, the autosampler WPS-3000TSL, and a gradient pump LPG-3400SD connected to a UV-Vis detector VWD-3100 (all Thermo Fisher Scientific, Waltham, MA, USA), equipped with an E150/2 Nucleodur HILIC column (hydrophilic interaction liquid chromatography; 150 × 2 mm, 3 μm particle size; MACHERY-NAGEL, Düren, Germany). The urea detection method took 30 min, and the column oven was heated to 40 °C. The mobile phase consisted of Millipore water (A) and 100% acetonitrile (B) with a flow rate of 0.3 mL min^− 1^. An analytical gradient was applied, starting with a ratio of 98% A and 2% B. After 2 min, the ratio of A was increased to 94.5% within 20 min and kept constant for 2 min. For the next 6 min, the ratio of A decreased to 80%, and afterwards the starting conditions of 98% A and 2% B were employed for 2 min. Peaks were detected at 205 nm using the UV-Vis detector.

#### Ammonium quantification

Filtered cell broth was diluted 1:20 with ultra-pure water, and a serial dilution ranging from 200 mg/L to 5 mg/L ammonium (NH_4_) was prepared as standard. A volume of 10 µL of the diluted samples or the calibration standard was pipetted into a 96-well plate. Afterward, 200 µL of a reagent solution consisting of 17.98 g L^− 1^ Na_3_PO_4_, 32 g L^− 1^ sodium salicylate, and 0.5 g L^− 1^ sodium nitroprusside was added to the sample. At last, 50 µL of 5% NaClO was added, and the plate was shaken for 0.5 min. After 12 min incubation time at room temperature, absorbance was measured at 685 nm in a Synergy Mix monochromator-based multi-mode microplate reader with Gen5 software (both from BioTek Instruments, Inc., Winooski, VT, USA) [113].

### Production of CO_2_-derived substrate mixtures

CO_2_-derived substrate mixtures are described in detail in the Additional File 1.

## Supplementary Information

Below is the link to the electronic supplementary material.

Supplementary Material 1.

## Data Availability

All data generated or analysed during this study are included in this published article and its supplementary information files.
