# Supplementary material for: Enhancing acetate-based biosurfactant production by Pseudomonas putida KT2440 through fermentation optimization
Source: Microb Cell Fact. 2026 Sep 29;25:195. doi: 10.1186/s12934-026-03129-0 (PMC13628881; doi:10.1186/s12934-026-03129-0)
Supplement: Supplementary file 1 [file 12934_2026_3129_MOESM1_ESM.pdf]

Additional files

Additional file 1

Producing CO_2-derived formate/acetate mixtures

All reagents and solvents were purchased from commercial suppliers. Procedures using air-sensitive compounds were carried out under an inert argon atmosphere (argon 4.8, *AirProducts, or Nippon Gases*) using the standard *Schlenk* technique or glovebox *(MBraun* LabMaster SP or *Sylatech* GB 1950-E). Glas equipment was dried at 85 °C overnight before use. For synthesis, the chemicals were degassed, and solvents were additionally dried over 3 Å molecular sieves before use. For catalytic reactions, the solvents were degassed *via* a glass frit by bubbling with argon for 30 minutes before use. Reaction gases, hydrogen 5.0 and carbon dioxide 4.6 were used without further purification.

High-pressure reactions

High-pressure reactions were carried out in stainless steel reactors built and maintained by the mechanical workshop of the Institute für Technische und Makromolekulare Chemie of RWTH Aachen University. The high-pressure reactors were equipped with a glass inlet and a magnetic stirring bar. The total inner volume with equipment was determined as 12.5 mL. To exclude oxygen from the system, in preparation for the experiments, vacuum was applied ($< 1 \times 10^{-2}$ mbar) followed by flushing with argon. This procedure was repeated at least three times. The high-pressure reactors, embedded in specially designed aluminum cones, were heated and stirred on heating plates (IKA-Werke GmbH & Co. KG, Staufen, Germany). The pressure was monitored with digital pressure sensors, and completion of the reaction was observed by constant pressure after pressure drop.

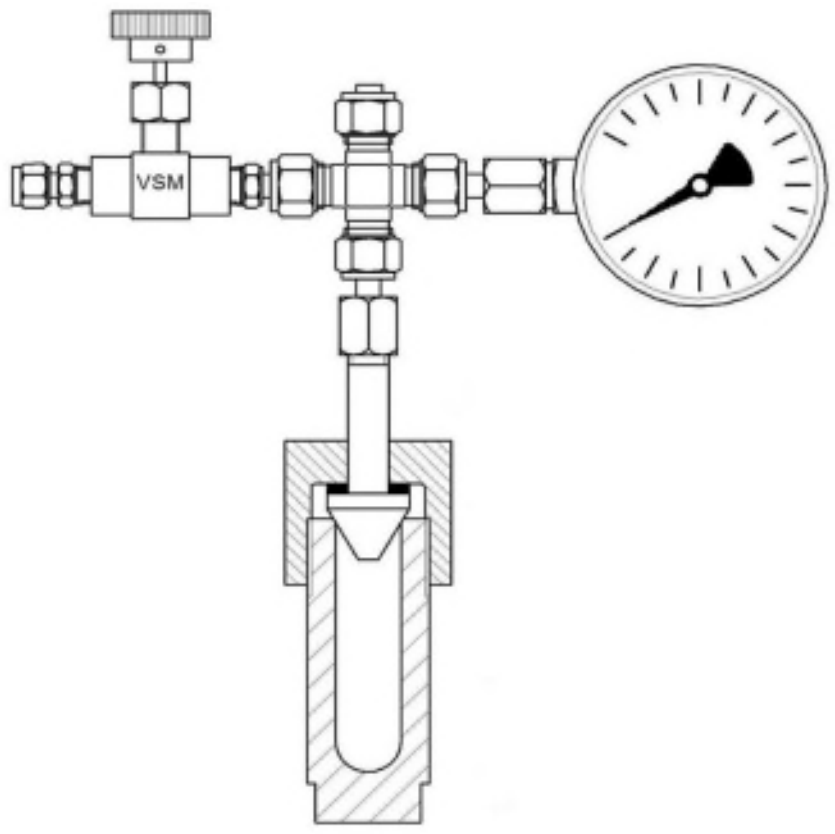

Additional file 1: Schematics of the used high-pressure reactors (digital manometers were used to record the pressure curves).

NMR analytic

^{1}H, ^{13}C, and ^{31}P NMR measurements were conducted at room temperature on a Bruker Ascend 400 (Bruker Corporation, Billerica, MA, USA) spectrometer equipped with "A BBO 400S1 BBF-H-D05 Z SP" or "CPP1.1 BBO 400S1 BB-H&F-D-05 Z XT" probe head. The spectra were processed and analyzed with MestReNova (version 14.2.3-29241) from Mestrelab Research S.L. The chemical shift δ is given in ppm, the coupling constant J in Hertz. The spectra were referenced using the residual solvent peaks for ^{1}H and ^{13}C NMR spectra ($CDCl_3$: δ_H = 7.26 ppm, δ_C = 77.2 ppm, D_2O: δ_H = 4.79 ppm) [1]. ^{31}P NMR spectra were referenced using aq. H_3PO_4 as an external standard. The multiplicities are denoted as s (singlet), d (doublet), t (triplet), quint (quintet), and m (multiplet). Quantitative analysis was conducted using DMSO as the internal standard for aqueous solutions and mesitylene for organic solutions.

Synthesis of *cis*-[$RuCl_2$(dppm)$_2$]

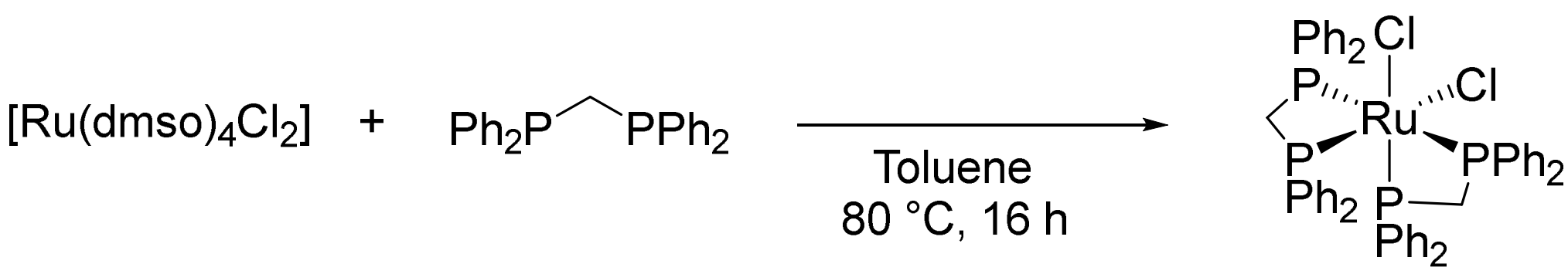

Additional file 1: Schematics of the reaction to synthesise the catalyst

The synthesis was performed according to literature procedure [2]. Into a *Schlenk*-tube [Ru(dmso)$_4$Cl$_2$] (242 mg, 0.5 mmol, 1 equiv.) and bis(diphenylphosphino)methane (384 mg, 1 mmol, 2 equiv.) were

weighed and dissolved in toluene (15 mL). The solution was stirred at 80 °C for 16 h. The progress of the reaction was controlled by ^{1}H and ^{31}P NMR spectroscopy in $CDCl_3$. After completion, the solvent was removed *via* cannula, and the residue was washed with *n*-pentane (3 x 10 mL) and Et_2O (2 x 10 mL). The resulting yellow solid was dried *in vacuo* at 120 °C (401 mg, 0.427 mmol, 85%).

^{1}H NMR ($CDCl_3$, 400 MHz): δ = 8.27 – 8.17 (m, 4H, Ar-*H*), 7.98 – 7.88 (m, 4H, Ar-*H*), 7.43 (td, *J* = 7.6 1.2 Hz, 4H, Ar-*H*), 7.36 (t, *J* = 7.3 Hz, 2H, Ar-*H*), 7.30 – 7.22 (m, 2H, Ar-*H*), 7.21 – 7.11 (m, 6H, Ar-*H*), 7.00 (td, *J* = 7.7, 1.9 Hz, 6H, Ar-*H*), 6.93 (td, *J* = 8.0, 4.1 Hz, 4H, Ar-*H*), 6.76 (td, *J* = 7.7, 1.6 Hz, 4H, Ar-*H*), 6.56 (td, *J* = 7.9, 4.2 Hz, 4H, Ar-*H*), 4.92 (ddd, *J* = 15.0, 6.4, 2.8 Hz, 2H, CH_2), 4.64 (dquint, *J* = 15.0, 4.3 Hz, 2H, CH_2) ppm.

^{31}P NMR ($CDCl_3$, 162 MHz): δ = −0.9 (t, $^{2}J_{PP}$ = 36.2 Hz, 2P, *P*Ph), −27.1 (t, $^{2}J_{PP}$ = 36.2 Hz, 2P, *P*Ph) ppm.

CO_2 hydrogenation in single batch experiments

The reactions were carried out as described above using a degassed, aqueous solution of sodium acetate (3 mL, 3M) and a solution of *cis*-[$RuCl_2(dppm)_2$]dissolved in degassed *n*-dodecanol (2 mL, 0.5 mM) as the catalyst phase. The autoclaves were sealed and pressurized with CO_2 (30 bar) and H_2 (60 bar) at room temperature. CO_2 pressurization was performed for 10 minutes under stirring to saturate the solution. This was confirmed by constant pressure over 1 min after closing the valve. The reaction was stirred at 60 °C until no further gas consumption was observable, indicated by constant pressure after an initial pressure drop. The autoclave was cooled in an ice bath for approximately 15 min, and the remaining pressure was released carefully. The aqueous and organic phases were removed, separated, and analyzed by ^{1}H NMR spectroscopy as described above.

Purification of CO_2-derived acetate

The reaction was carried out as described above in a 50 mL autoclave with [$PdCl_2$(dppe)] (3.60 mg, 6.25 μmol), dppe (4.98 mg, 12.5 μmol, NaOMe (58.1 mg, 1.01 mmol), and NaI (2.19 g, 14.6 mmol) being weighed into the autoclave. Methyl formate (19.6 g, 326 mmol) was added, and the reaction

was stirred at 173 °C for 120 h. The autoclave was cooled in an ice bath for approximately 15 min, and the remaining pressure was released carefully. The reaction solution was filtered and purified by vacuum distillation. The product acetic acid was transformed into its corresponding sodium salt by stoichiometric reaction with NaOMe in MeOH. The solvent was removed under reduced pressure, and the product was dried for several hours at 85 °C to give a white solid. The obtained sodium acetate was successfully employed as stabilizing base in the ruthenium-catalyzed CO_2 hydrogenation to formic acid.

Additional file 2

Oxygen transfer rate profiles and physiological parameters obtained during comparative microtiter plate cultivations

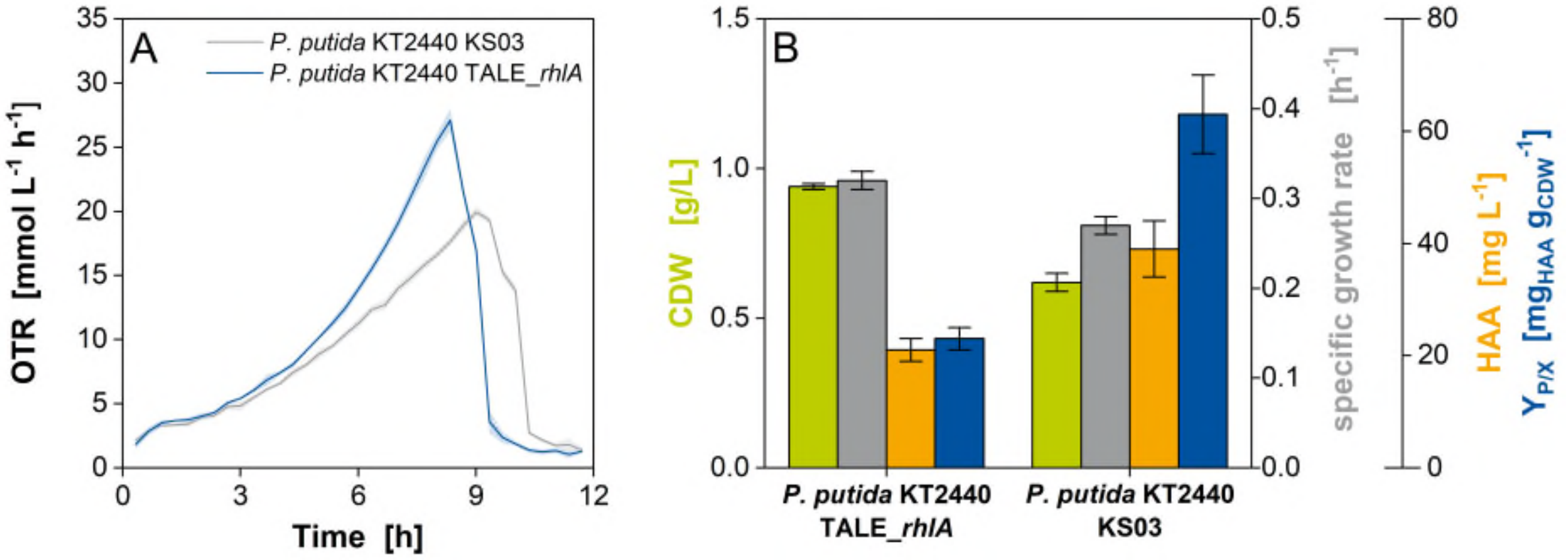

Additional file 2: Comparison of physiological characteristics of *P. putida* TALE_*rhlA* and *P. putida* KS3 during acetate cultivation. (A) OTR profiles over time, (B) CDW at the end of cultivation (green bars), specific growth rates (grey bars), HAA concentrations at the end of cultivation (orange bars), and product-to-biomass yields (dark blue bars). Cultivation conditions: MSM medium containing 5 g L^{-1} acetate, 2 g L^{-1}ammonium sulfate, and 4x buffer, T = 30 °C; pH_{start} = 7.0, OD_{start} = 0.1, V_{fill} = 0.5 mL in a 96-Well Plate, N = 300 rpm, d = 50 mm, Measurement of OTR: microTOM Kuhner System, n = 4. Oxygen transfer rate profiles are shown as mean values with standard deviations indicated by shaded areas (n = 4). Maximum HAA concentration, CDW, specific growth rate, and product-to-biomass yield are presented as mean ± standard deviation of four replicates (n = 4). CDW = cell dry weight, $Y_{P/X}$ = product-to-biomass yield.

Additional Table 2: Physiological parameters and performance indicators of *P. putida* KT2440 TALE_*rhlA* and *P. putida* KT2440 KS3 in small-scale cultivation.

Strain		***P. putida* KT2440 TALE_*rhlA***	***P. putida* KT2440 KS3**
µ	[h^{-1}]	0.32 ± 0.01	0.27 ± 0.01
OTR_{max}	[mmol L^{-1} h^{-1}]	27.1 ± 0.8	19.9 ± 0.4
CDW	[g L^{-1}]	0.94 ± 0.01	0.62 ± 0.03
$c_{max, HAA}$	[mg L^{-1}]	21 ± 2	39 ± 5
$Y_{X/S}$	[g_{CDW} g_{Ac}^{-1}]	0.19 ± 0.01	0.12 ± 0.01
$Y_{P/S}$	[mg_{HAA} g_{Ac}^{-1}]	4 ± 1	8 ± 1
$Y_{P/X}$	[mg_{HAA} g_{CDW}^{-1}]	23 ± 2	63 ± 7

P. putida TALE_*rhlA*: *P. putida* KT2440 evolved for acetate tolerance equipped with gene for HAA production, *P. putida* KS3: *P. putida* KT2440 wild-type strain equipped with gene for HAA production. Parameters: µ = specific growth rate during exponential phase, OTR_{max} = maximal OTR reached during cultivation, CDW = cell dry weight at the end of cultivation, $c_{HAA, max}$ = maximal HAA concentration at the end of cultivation, $Y_{X/S}$ = Biomass-to-substrate yield, $Y_{P/S}$ = Product-to-substrate yield, $Y_{P/X}$ = Product-to-biomass yield. Data were obtained from comparative microtiter plate cultivation under identical cultivation conditions. Values represent the mean ± standard deviation of four biological replicates (n = 4).

Additional file 3

pH- and DO-coupled feeding strategy prevents carbon limitation, but affects pH value

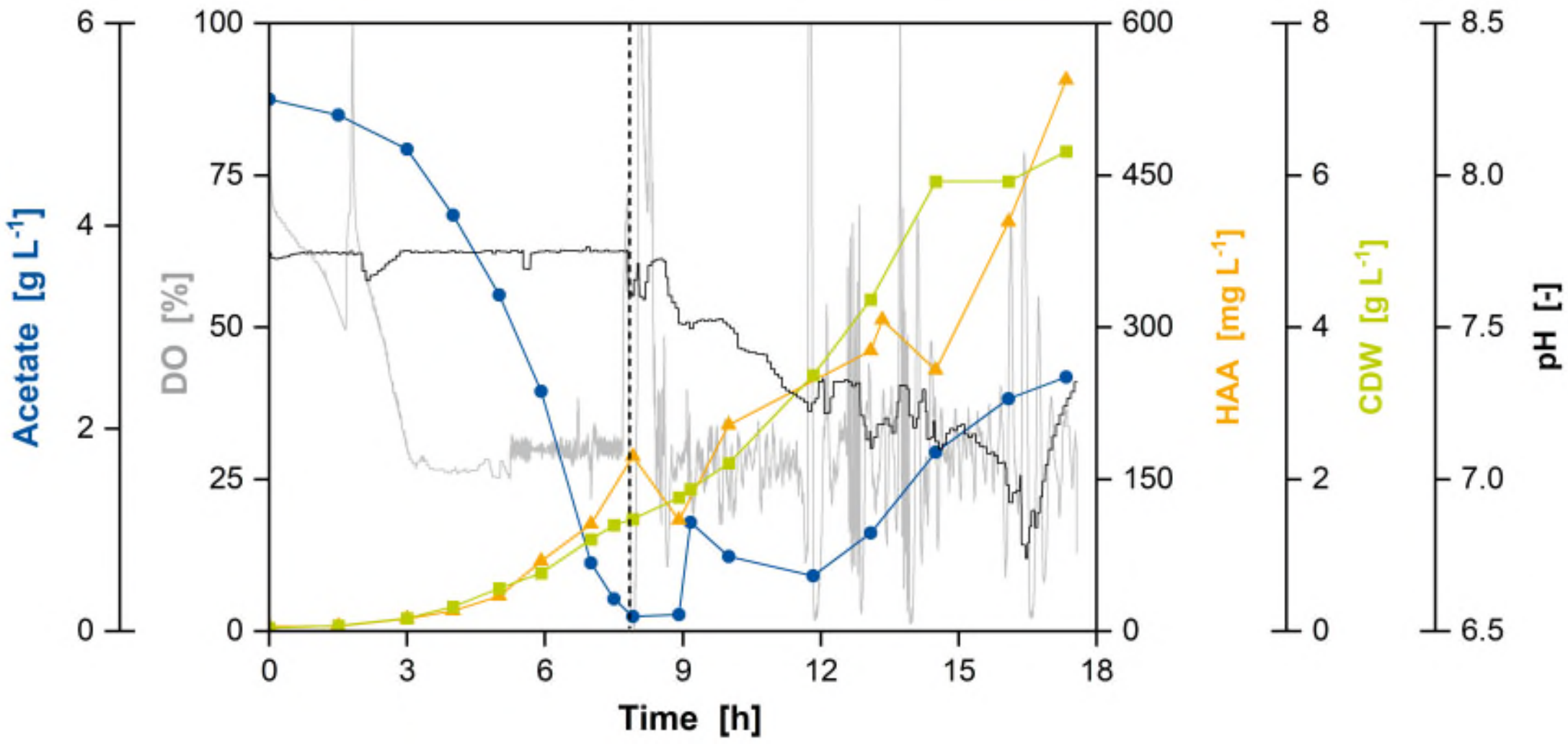

Additional file 3: pH- and DO-coupled feeding strategy with ammonium sulfate as nitrogen source using *P. putida* KS3. Membrane-aerated fed-batch cultivation with acetate concentration (blue line, circles), DO (light grey lines), HAA concentration (orange line, triangles), CDW (light green lines, squares), and pH value (black lines). Cultivation conditions: MSM medium containing 5 g L^{-1} acetate and 1x buffer, nitrogen source: 2 g L^{-1} ammonium sulfate, 3 L stirred-tank bioreactors with 2 L filling volume, T = 30 °C, N = 100 - 350 rpm (cascaded to maintain a DO ≥ 30% with subsequent increase of TMP to 0.3 bar and supplementation of pure oxygen (21%-100%)), F_{Air} = 1 L min^{-1}, OD_{start} = 0.2, pH = 7.8, pH value maintained by 4 M H_2SO_4 (batch phase). Feed: glacial acetic acid, Pulses: 1.5 g Na-acetate, CDW: cell dry weight, DO: dissolved oxygen, n = 1.

Additional file 4

Bubble-aerated pH-coupled feeding strategy with urea as nitrogen source

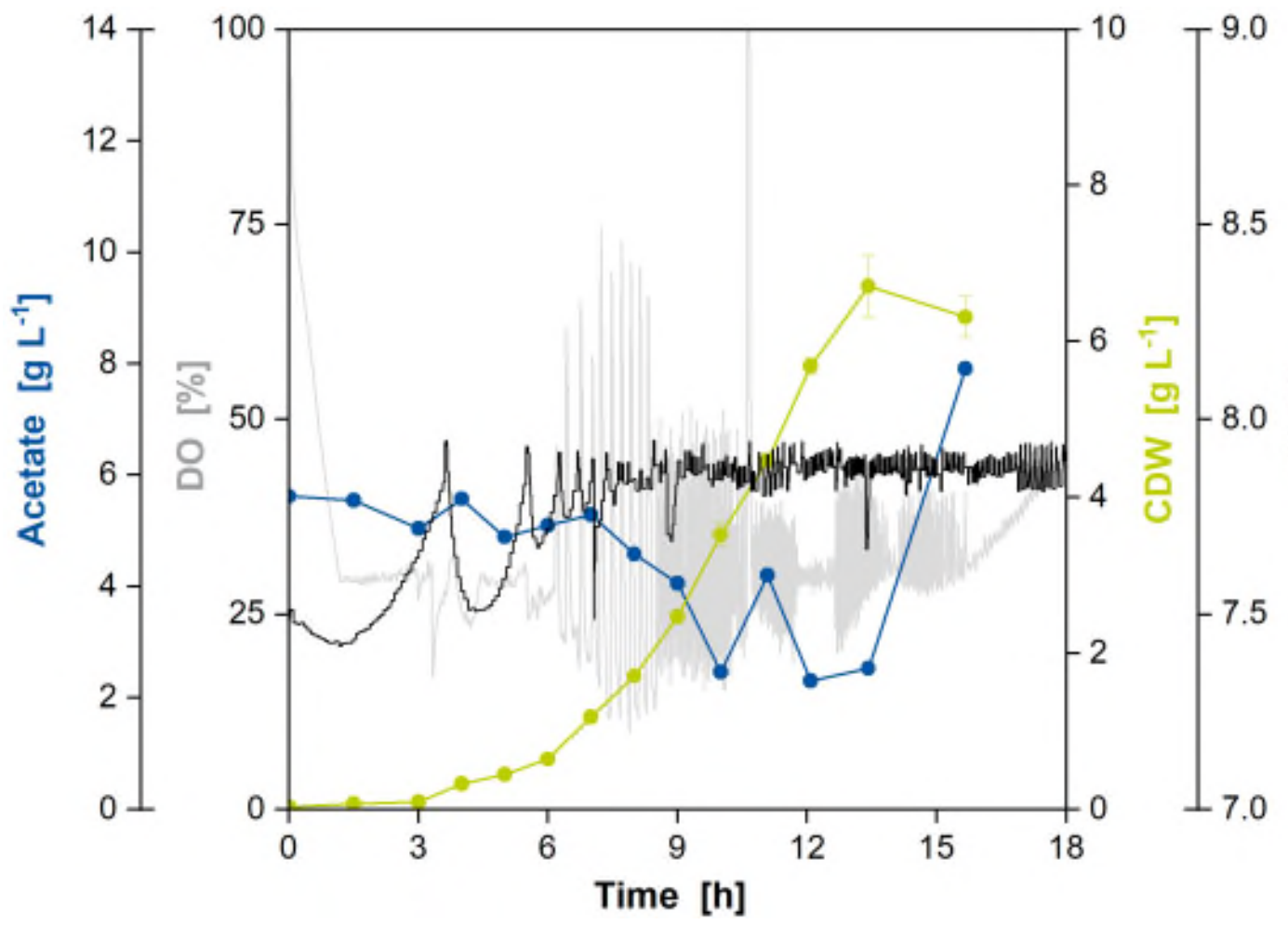

Additional file 4: pH-coupled feeding-strategy with urea as nitrogen soure using *P. putida* KT2440. Bubble-aerated fed-batch cultivation with acetate concentration (blue line, circles), DO (light grey lines), CDW (green lines, squares), and pH value (black line) over time. Cultivation conditions: MSM medium containing 5 g L^{-1} acetate and 1x buffer, nitrogen source: 0.9 g L^{-1} urea, 1.3 L stirred-tank bioreactors with 0.5 L filling volume, T = 30 °C, N = 350 - 1,000 rpm (cascaded to maintain a DO ≥ 30%), F_{Air} = 0.5 L min^{-1}, OD_{start} = 0.2, pH = 7.8, pH value maintained with glacial acetic acid containing 25 g L^{-1} urea and pH value of 2.0, CDW: cell dry weight, DO: dissolved oxygen, n = 1.

Additional file 5

CO_2-derived acetate mixture is not compatible with *P. putida* KT2440

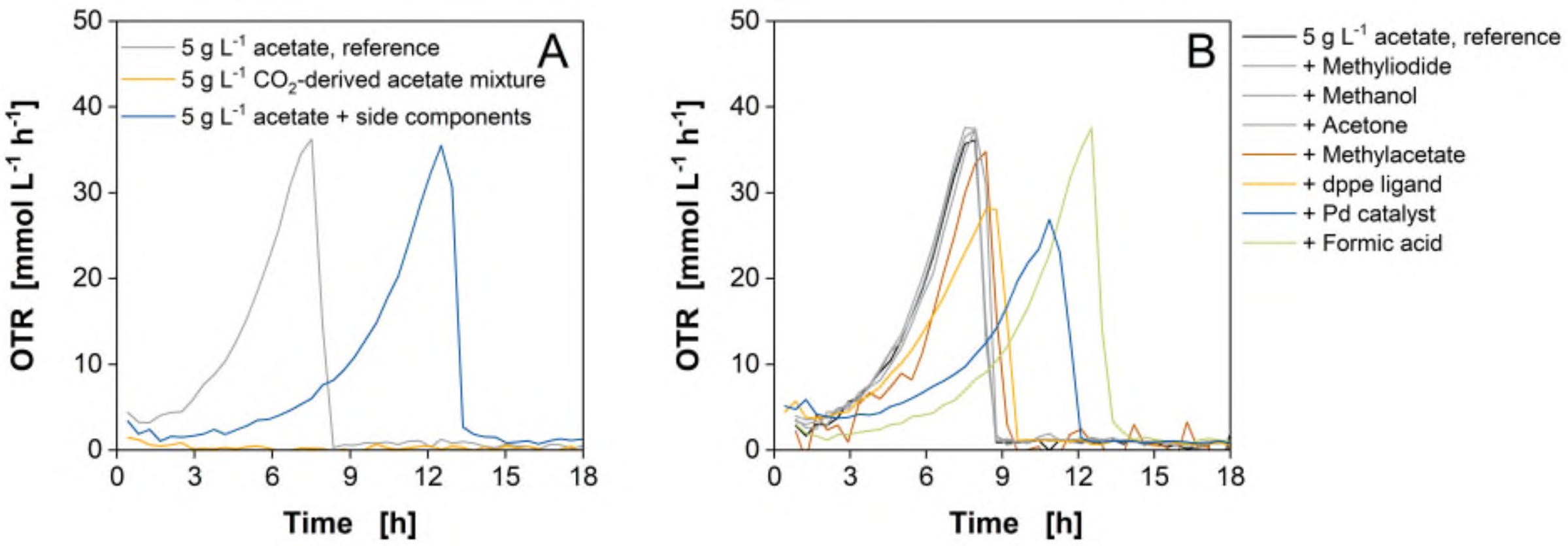

Additional file 5: Investigation of CO_2-derived acetate mixture using *P. putida* TALE_*rhlA* in shake flasks. Growth (A) and biocompatibility test (B) on chemically derived acetate substrate mixture. Synthetic mix (A): supplementation of most prominent side components (methyl acetate, ethyl acetate, formic acid, methanol) according to [3]. Biocompatibility tests included the addition of catalyst components and ligands, as well as all side components, in single cultivations (B). Only components that influence growth are shown with colors. Growth on 5 g L^{-1} acetate or 5 g L^{-1} chemically derived acetate. MSM Medium, T = 30 °C, pH_{start} = 7.0, OD_{start} = 0.1, V_L = 25 mL in 500 mL shake flasks, n = 300rpm, n = 1 (biocompatibility tests), n = 2 (ref/chem. acetate); Measurement of OTR: TOM System, Kuhner Shaker. Error bars represent the standard deviation of the mean.

1. Fulmer GR, Miller AJM, Sherden NH, Gottlieb HE, Nudelman A, Stoltz BM, et al. NMR chemical shifts of trace impurities: Common laboratory solvents, organics, and gases in deuterated solvents relevant to the organometallic chemist. Organometallics. 2010;29(9):2176-9. https://doi.org/10.1021/om100106e.
2. Scott M, Blas Molinos B, Westhues C, Franciò G, Leitner W. Aqueous biphasic systems for the synthesis of formates by catalytic CO_2 hydrogenation: Integrated reaction and catalyst separation for CO_2-scrubbing solutions. ChemSusChem. 2017;10(6):1085-93. https://doi.org/10.1002/cssc.201601814.
3. Jürling-Will P, Botz T, Franciò G, Leitner W. A "Power-to-X" route to acetic acid via palladium-catalyzed isomerization of methyl formate. ChemSusChem. 2022;15(16):e202201006. https://doi.org/10.1002/cssc.202201006.
